# Enzymatic and synthetic regulation of polypeptide folding

**DOI:** 10.1039/d3sc05781j

**Published:** 2024-01-26

**Authors:** Takahiro Muraoka, Masaki Okumura, Tomohide Saio

**Affiliations:** a Department of Applied Chemistry, Graduate School of Engineering, Tokyo University of Agriculture and Technology Koganei Tokyo 184-8588 Japan muraoka@go.tuat.ac.jp; b Kanagawa Institute of Industrial Science and Technology (KISTEC) Kanagawa 243-0435 Japan; c Frontier Research Institute for Interdisciplinary Sciences, Tohoku University Sendai Miyagi 980-8578 Japan okmasaki@tohoku.ac.jp; d Division of Molecular Life Science, Institute of Advanced Medical Sciences, Tokushima University Tokushima 770-8503 Japan saio@tokushima-u.ac.jp

## Abstract

Proper folding is essential for the biological functions of all proteins. The folding process is intrinsically error-prone, and the misfolding of a polypeptide chain can cause the formation of toxic aggregates related to pathological outcomes such as neurodegenerative disease and diabetes. Chaperones and some enzymes are involved in the cellular proteostasis systems that assist polypeptide folding to diminish the risk of aggregation. Elucidating the molecular mechanisms of chaperones and related enzymes is important for understanding proteostasis systems and protein misfolding- and aggregation-related pathophysiology. Furthermore, mechanistic studies of chaperones and related enzymes provide important clues to designing chemical mimics, or chemical chaperones, that are potentially useful for recovering proteostasis activities as therapeutic approaches for treating and preventing protein misfolding-related diseases. In this Perspective, we provide a comprehensive overview of the latest understanding of the folding-promotion mechanisms by chaperones and oxidoreductases and recent progress in the development of chemical mimics that possess activities comparable to enzymes, followed by a discussion of future directions.

## Abstract 2

This perspective focuses on the latest understanding of the folding-promotion mechanisms by chaperones and oxidoreductases and recent progress in the development of chemical mimics that possess activities comparable to enzymes.

## Graphical abstract

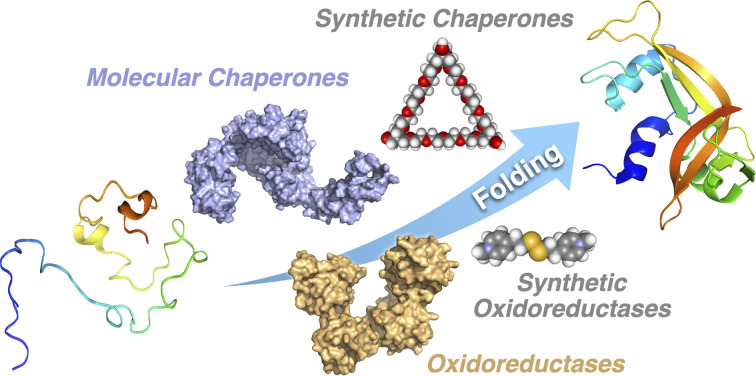

## Introduction

For all proteins to decide their individual biological functions, the nascent polypeptide chain must be co- or post-translationally folded into its unique three-dimensional structure called a native conformation.^1–3^ Polypeptide folding is intrinsically error-prone but must proceed along a defined folding trajectory within the energy landscape under thermodynamic and kinetic control.^4–6^ Several stresses, such as Ca^2+^ depletion and reactive oxygen species generation, disrupt the folding pathway, leading to the formation of misfolded or non-native conformations of the polypeptide chain.^7^ Misfolded protein species often aggregate *via* intermolecular contact between exposed hydrophobic amino acid residues.^8^ Because aggregated polypeptides are highly toxic to cells and therefore adversely impact human health,^9,10^ cells possess sophisticated protein homeostasis mechanisms, such as proteostasis, systems that assist protein folding *via* chaperones^11^ and related enzymes, which diminish the risks associated with polypeptide aggregation.^12^ Loss-of-function of chaperones and related enzymes can thus result in serious pathological outcomes, such as neurodegeneration and diabetes.^13–16^ Therefore, a more complete understanding of chaperone-mediated and enzymatic proteostasis systems could provide important clues to develop means of preventing protein misfolding-related pathologies involving biological approaches to reinforce the proteostasis system^17^ and chemical regulation to supplement impaired proteostasis activities.^18^

Over the last several decades, researchers have gained substantial insight into proteostasis networks in the cytosol. In the highly concentrated cellular environment, in which the protein concentration in the cytosol can reach 300–400 g L^−1^,^19^ unfolded or misfolded polypeptides are prone to aggregate. Several molecular chaperones function to help maintain proteostasis in the crowded intracellular environment.^17,20^ Representative functions of molecular chaperones include anti-aggregation, foldase, and holdase activities. Anti-aggregation activity prevents newly synthesized nascent polypeptides from aggregating with other molecules in the cell. Foldase activity assists in and promotes the folding of the polypeptide chain to acquire the proper native conformation.^21,22^ Holdase activity keeps the polypeptide chain in an extended conformation needed for translocation through membrane channels.^20,23^ Molecular chaperones such as members of the heat-shock protein (Hsp) family capture client unfolded polypeptides and provide a protected folding environment that inhibits aggregation.^24^ Chaperones recognize immature or aggregation-prone polypeptides by binding to exposed hydrophobic segments that are buried in the core of the native conformation. The client polypeptide binding and releasing cycle is driven by ATP hydrolysis and the exchange of resultant ADP for ATP.^25^

In addition to the regulation of protein folding, recent studies have unveiled the functional importance of molecular chaperones in regulation of protein assembly, especially biological liquid–liquid phase separation (LLPS). For instance, Hsp27 and Hsp40 have been reported to regulate LLPS of fused in sarcoma (FUS).^26,27^ Not only these heat shock proteins, but also a nuclear import receptor Kapβ2 has been reported to regulate FUS LLPS.^28^ Given the fact that FUS and its LLPS regulation are related to neurodegenerative diseases especially amyotrophic lateral sclerosis (ALS), the mechanistic understanding of regulation and dysregulation of FUS LLPS has been anticipated in the life science research field.^29^ Some chaperones are also known to work cooperatively with LLPS proteins. One important example is seen in the nucleolus, a membrane-less organelle formed through LLPS of nucleus proteins: it has been proposed that the nucleolus has chaperone-like properties and incorporates the misfolded proteins associated with Hsp70 to preserve their reversible assembling properties.^30^

Nearly one-third of nascent polypeptides enter the endoplasmic reticulum (ER), where folding-assisting enzymes such as molecular chaperones and oxidoreductases promote efficient folding and facilitate formation of the native conformation by preventing aggregation and promoting the formation of disulfide bonds.^31^ Intramolecular disulfide bonds contribute to the stabilization of local or global conformations by decreasing the configurational entropy of the polypeptide chain.^32–37^ As the formation of erroneous or non-native disulfide bonds can cause misfolding and aggregation of polypeptides, it is important to accelerate the isomerization of non-native disulfide bonds to facilitate correct oxidative folding.^38,39^ In the case of intermolecular disulfide bonds involved in folding/assembly such as human chorionic gonadotropin^40^ and fibrinogen,^41^ non-native disulfide bonds must be effectively cleaved and exchanged to intermolecular native disulfide bonds during the folding/assembly process to avoid forming misfolding and aggregates.^42^ Protein disulfide isomerase (PDI) and over 20 members of the PDI family are the most abundant oxidoreductases in the ER and catalyze the formation and isomerization of intra- or inter-molecular disulfide bonds.^43–45^ PDI plays a central role in the oxidative protein folding associated with disulfide bond formation by coupling redox-active functions with chaperone activity.^46,47^

Inspired by the chaperone-mediated and enzymatic promotion of protein folding in cells, chemists have developed synthetic mimics of chaperones and oxidoreductases. Small molecules and polymeric materials that interact with unfolded polypeptides to enhance their water solubility show chaperone-like functions by preventing aggregation. Thiol-based redox-active compounds, as synthetic mimics of the catalytic center of PDI, promote disulfide bond shuffling in polypeptide chains and facilitate the formation of the native conformation. Such chemical chaperones could be useful for not only the production of functional and pharmaceutical proteins but also the recovery and reinforcement of collapsed proteostasis activities as a therapeutic approach.^48^ Recent progress afforded a synthetic oxidoreductase mimic in which the protein folding activity is comparable to that of enzymes.^38^

Folding assistance has been studied intensely for almost 50 years, and such studies are important not only for enhancing fundamental scientific understanding but also understanding protein misfolding- and aggregation-related pathophysiology.^49^ In this Perspective, we provide an overview of the latest understanding of the biological folding-promotion mechanisms of chaperones and oxidoreductases. Recent synthetic approaches inspired by biological folding-promotion processes are also described, with a discussion of several lines of evidence supporting *de novo* chemical chaperone strategies potentially useful for therapeutic applications.

## Unraveling the mechanism of protein folding and holding by molecular chaperones

### Structural investigations of chaperone–client complexes

The mechanisms by which molecular chaperones alter the folding properties of client proteins have been extensively studied. One approach is to determine the structures of chaperones. Structural studies using X-ray crystallography and cryo-electron microscopy (cryo-EM) have revealed a number of crystal structures of molecular chaperones.^50^ The distinct shapes of molecular chaperones enable researchers to surmise how the molecular chaperones function. For instance, the chamber shapes of GroEL/ES^51^ and TRiC/CCT^52^ suggest that these chaperones isolate the immature protein for folding without interruption *via* nonspecific interactions with other proteins. Moreover, structures of Hsp90 dimers in various conformations that depend on nucleotide binding and hydrolysis^53,54^ suggest that Hsp90 “pinches” the client protein. As indicated above, the structures of chaperones have provided insights into their mechanisms of action. However, not many structures of chaperone–client complexes are available, especially for those in which the client protein is in an unfolded state. Structural analyses can be difficult due to the mobility of the unfolded client protein and weak interactions between the client protein and molecular chaperones, as these factors hinder crystallization and cryo-EM analyses.^29,55–60^

**Fig. 1:**
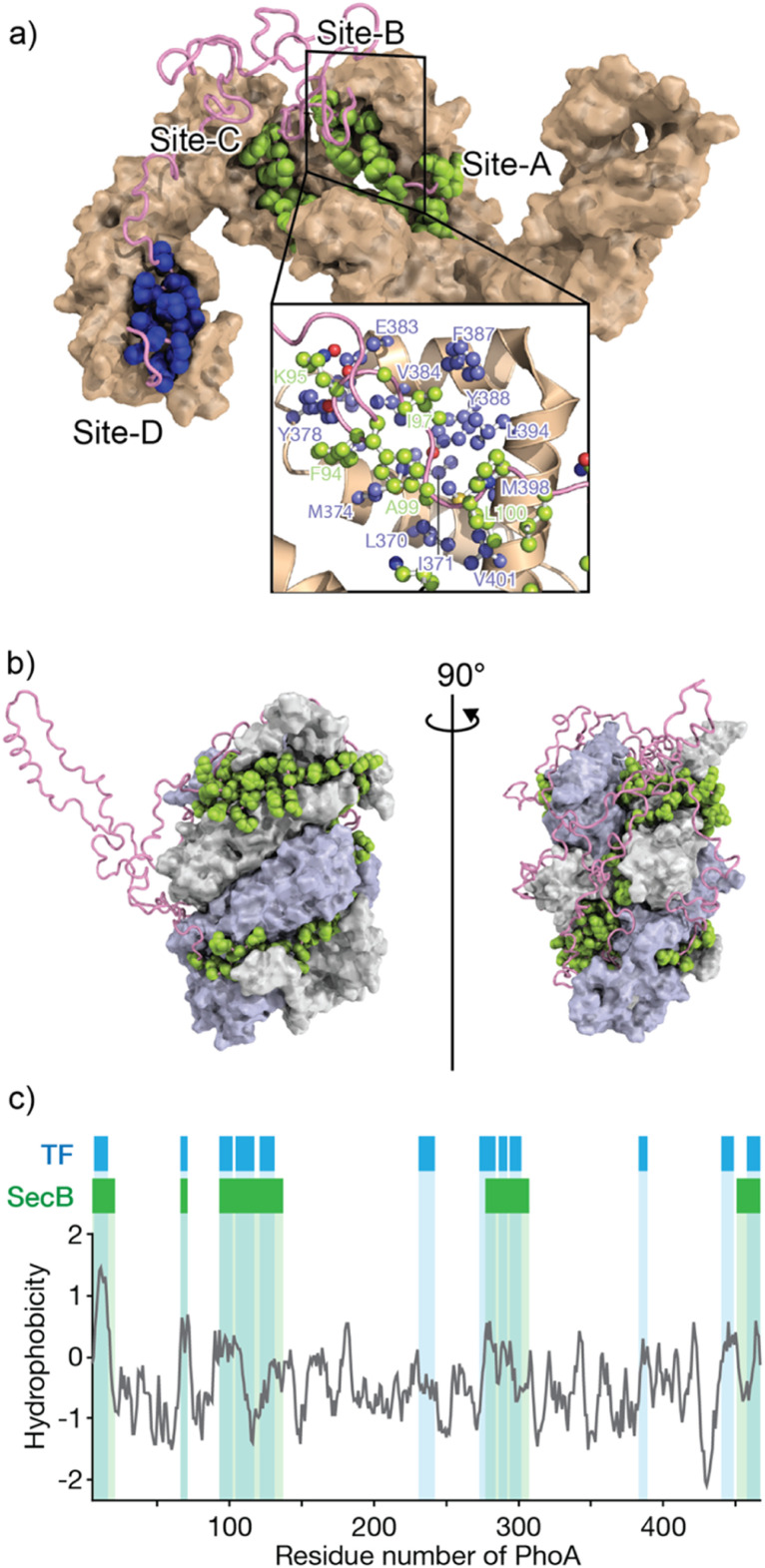
Structures of TF and SecB chaperones capturing an unfolded protein. (a) Structure of the TF chaperone in complex with unfolded client protein PhoA1-150 (PDB ID: 2MLY).^55^ TF is represented as a brown surface model. PhoA is represented as a pink chain, with the amino acids recognized by TF shown as spheres. The inserted image represents the expanded view of TF site-B, in which TF is represented as a brown ribbon with the amino acid residues involved in recognition of the client protein shown as a ball and stick model colored blue. (b) Structure of SecB in complex with unfolded client protein PhoA (PDB ID: 5JTL). The SecB tetramer is represented as a surface model colored gray and pale blue. PhoA is represented as a pink chain, with the amino acids recognized by SecB shown as spheres. The amino acid residues involved in the interaction were indicated. (c) Hydrophobicity plot of PhoA with the regions recognized by TF and SecB highlighted blue and green, respectively. A hydrophobicity score (Roseman algorithm, window = 9) greater than zero denotes increasing hydrophobicity.

Solution nuclear magnetic resonance (NMR) spectroscopy, on the other hand, has been extensively used in studies of chaperone–client complexes because of the advantages of NMR in structural studies of dynamic and weak protein complexes. Although the application of conventional NMR analysis to protein structural studies has been generally limited to relatively small proteins <20–30 kDa, recent advances in NMR techniques, including hardware, isotope labeling,^56,57^ and pulse sequences,^58^ have significantly extended the size limit. With these recently developed NMR techniques, NMR structural studies of proteins >100 kDa, including molecular chaperones, are now feasible.^29,59,60^ One of the first reported structures of a molecular chaperone in complex with an unfolded client protein was that of trigger factor (TF) chaperone in complex with unfolded alkaline phosphatase (PhoA) (Fig. 1a).^55^ TF is a molecular chaperone in the bacterial cytosol and consists of three structural domains: a ribosome-binding domain, substrate-binding domain, and peptidylprolylisomerase domain.^52,53^ TF recognizes unfolded client proteins and prevents their aggregation and promotes their folding or translocation. The structure of the TF–PhoA complex shows that TF captures the hydrophobic stretches of the unfolded client protein at four distinct binding sites (Fig. 1a). Each client-binding site of TF recognizes 5 to 10 amino acid residues of the client protein. However, the regions recognized by TF are sparsely located within the PhoA amino acid sequence, resulting in one TF molecule accommodating 100 to 150 residues of the client protein. Thus, the TF chaperone recognizes multiple short segments of the client protein.

The structure of SecB in complex with unfolded PhoA was also determined by solution NMR spectroscopy.^61^ Analysis of the structure showed that the SecB tetramer forms a disc-shaped structure surrounded by unfolded PhoA (Fig. 1b). In contrast to TF, which accommodates 100 to 150 residues of unfolded PhoA, the SecB tetramer accommodates all 471 amino acid residues of PhoA. Interestingly, although the overall shapes of the complexes are distinct, the two chaperones have several features in common. First, both chaperones include multiple distinct binding sites for client proteins. Second, the regions of PhoA recognized by the chaperones are quite similar in both cases, as the regions recognized consist of hydrophobic amino acid residues (Fig. 1c). Furthermore, it should be emphasized that the client protein is kept unfolded in complex with these chaperones (Fig. 1a and b). Given that the hydrophobic regions in the client protein are folded into the hydrophobic core in the native protein, recognition of the hydrophobic regions of the client protein by the chaperones suggests that binding to the molecular chaperones suppresses folding. Indeed, SecB exhibits strong holdase activity and suppresses folding of the client protein. In contrast, although TF is known to exhibit holdase activity, it can also function as a foldase to enhance the folding rate and yield.

### Kinetics–activity relationships in chaperone function

**Fig. 2:**
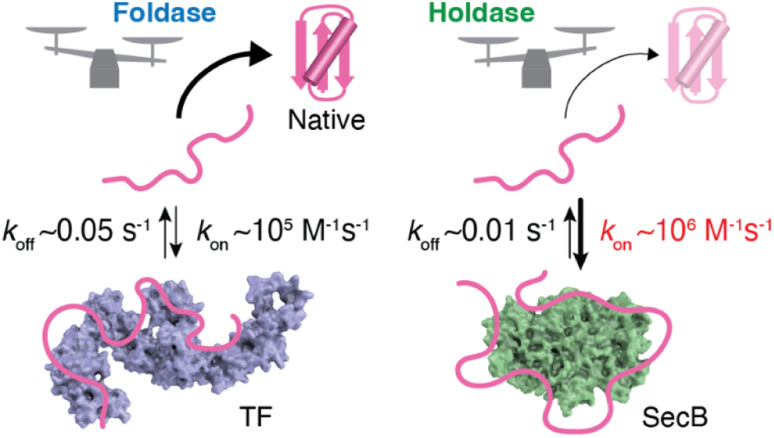
Schematic representation of the kinetics–activity relationship seen in TF and SecB. SecB has a more rapid *k*_on_ for the client protein than TF and thus shows stronger holdase activity. The structures of TF and SecB are drawn using PDB coordinates of 1W26 (ref. 64) and 1QYN,^65^ respectively.

As described in the previous section, structural information regarding molecular chaperones in complex with client proteins has revealed the mechanism of client protein recognition. However, the mechanism of how the chaperones alter the folding properties of the client proteins remained reclusive. More specifically, both SecB, which is believed to function exclusively as a holdase, and TF, which is known to function as both a foldase and holdase depending on the conditions, share essentially the same mechanism of client protein recognition, as revealed by structural analyses. To understand the mechanism underlying the distinct functional characteristics of the foldase and holdase activities, a view of binding kinetics is important. The structural and dynamics studies highlighted that the chaperone–client complex is not static but rather dynamic, undergoing rapid on–off exchange (Fig. 2).^55^ In addition to TF and SecB, it has been reported that Hsp40 recognizes unfolded client protein in a dynamic manner.^62^ Thus, further exploration of this dynamic property could play a key role in determining the mechanisms underlying the function of molecular chaperones. Interestingly, kinetic measurements of the binding of the chaperones and unfolded client proteins revealed that TF and SecB exhibit distinct binding kinetics for unfolded maltose-binding protein (MBP) as a client, with SecB exhibiting a more rapid *k*_on_ and slower *k*_off_ for the client than TF.^61^ Although *k*_off_ also contributes to the functional characteristics of molecular chaperones, it was proposed that differences in *k*_on_ explain the differences in holdase/foldase activities. As indicated by the structures of the TF–PhoA and SecB–PhoA complexes, both chaperones capture hydrophobic stretches of the client protein to hold it in an unfolded state. In contrast, given the dynamic nature of the chaperone–client complex, the complex is not static but undergoes on–off exchange. Here, the client protein is sometimes released from the molecular chaperone for a time to allow for spontaneous folding. However, if the molecular chaperone has a more rapid *k*_on_, the client protein is recaptured after a short period, and accordingly, the client protein is not given enough time for folding. Thus, chaperones exhibiting more rapid *k*_on_ values would be expected to exert stronger holdase activity. It should be noted that the association rate is determined by the product of *k*_on_ and concentration, suggesting that holdase activity is stronger at higher concentrations of the molecular chaperone. Indeed, a previous study showed that the holdase activity of SecB becomes stronger as its concentration increases.^61^ These observations and discussion regarding the relationship between activity and chaperone concentration highlight the importance of the cellular concentration of a molecular chaperone in determining its functional properties. Another point that must be considered is the intrinsic folding rate of the client protein. If the client protein folds quickly enough after being released from the molecular chaperone, it can “escape” binding to the molecular chaperone. On the other hand, if the client protein exhibits a slower spontaneous folding rate, a molecular chaperone with a moderate *k*_on_ value would be expected to also function as a holdase. Indeed, TF, which exhibits a moderate *k*_on_ value, functions as a holdase for an MBP mutant with a low intrinsic folding rate.^61^ This trend demonstrates that the effects of molecular chaperones can differ for each client protein. Thus, the effects of the molecular chaperones on the folding of client proteins are determined by the balance between the binding kinetics and intrinsic folding rate of the client protein.^63^

This kinetics–activity relationship is consistent with the “kinetic partitioning” theory proposed in the 1990s to explain the mechanism of selection of the client protein of SecB for the secretary pathway.^23^ As indicated by the examples described above, recognition of the sequence of the client protein by molecular chaperones is promiscuous, whereas the kinetic properties are variable. Promiscuous recognition is important for molecular chaperones to support the folding of many types of client proteins. The kinetics of promiscuous binding enable molecular chaperones to exhibit functional variety and client specificity. It is currently difficult to declare the general criteria to distinguish foldase/holdase because of the limited number of examples for kinetic parameters. Further kinetic studies for multiple chaperones and client proteins with varying folding rates should be anticipated for comprehensive understanding of the activity–kinetics relationship.

### Functional importance of oligomerization of molecular chaperones

**Fig. 3:**
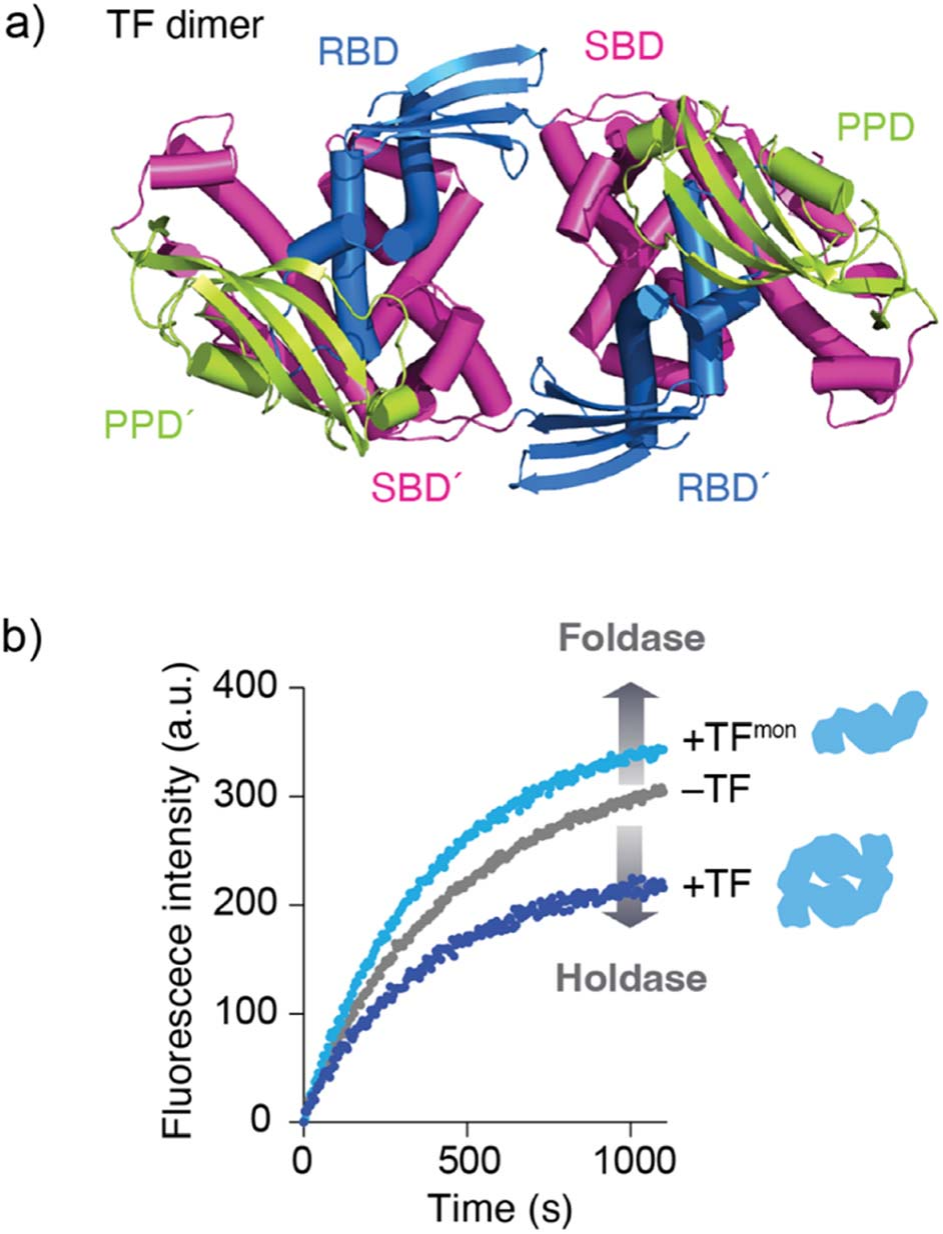
Structure and activity of the TF dimer. (a) Structure of the TF dimer (PDB ID: 6D6S).^59^ (b) Refolding assay of MBP showing foldase and holdase activities of the TF monomer and dimer. RBD; ribosome-binding domain, SBD; substrate-binding domain, and PPD; peptidylprolylisomerase domain.

Although the mechanism by which binding kinetics are modulated is poorly understood, recent studies have highlighted the importance of oligomerization of molecular chaperones in kinetic modulation. One representative example has been shown for TF. In solution, TF exists in equilibrium between monomers and dimers, with a *K*_D_ of ∼2 μM. Here, NMR analysis showed that TF forms a dimer in an anti-parallel, head-to-tail manner (Fig. 3a).^59^ Comparison of the activity between the TF dimer and TF monomeric mutant showed that the TF dimer exhibits stronger holdase activity than the monomer (Fig. 3b). Consistent with this trend in holdase activity, the TF dimer has a more rapid *k*_on_ than the monomer. Coupled with dimerization, this modulation of activity can be important for TF to exert multiple functions in the cell. For example, TF functions as (1) a ribosome-associated molecular chaperone as a monomer to guide newly synthesized nascent protein chains out of the ribosome,^52,57^ (2) a cytosolic molecular chaperone as a dimer to help facilitate folding of the newly synthesized protein and prevent its aggregation or misfolding, and (3) a chaperone in the protein translocation machinery consisting of SecA and SecB to maintain the client protein in an unfolded state and transfer it to SecB.^58^ At different locations and under different situations, the functional properties of TF change. One hypothesis suggests that this functional modulation is coupled with kinetic modulation due to the oligomeric states of TF and binding to other molecular chaperones, although further studies will be needed. Activity modulation coupled with oligomerization is also seen for the TF in *Thermus thermophilus* (*Tt*TF), which is activated through zinc-induced oligomerization.^66,67^ The *in vitro* biochemical study identified that the zinc activates the holdase activity of *Tt*TF, in which the refolding of green fluorescent protein (GFP) was arrested in the presence of *Tt*TF and zinc ions.^67^ The subsequent study identified that zinc binding capacity is specific to *Tt*TF among the tested TF species from *Thermus thermophilus*, *Thermotoga maritima*, and *Escherichia coli.*^66^ Although the detailed mechanism remained to be elucidated, the study unveiled that zinc binding induces a change in the secondary structures and oligomerization of *Tt*TF. Activity modulation coupled with oligomerization has been reported not only for TF but also other molecular chaperones, including Skp,^68^ HSP16.6 from the cyanobacterium *Synechocystis*,^69^ and DNAJB6.^70,71^ These examples suggest that oligomerization of molecular chaperones plays a key role in determining their functional specificity through promiscuous binding.

## ER-resident PDI family members as disulfide-catalysts to ensure oxidative protein folding fidelity

### PDI family-related diverse misfolding diseases

**Fig. 4:**
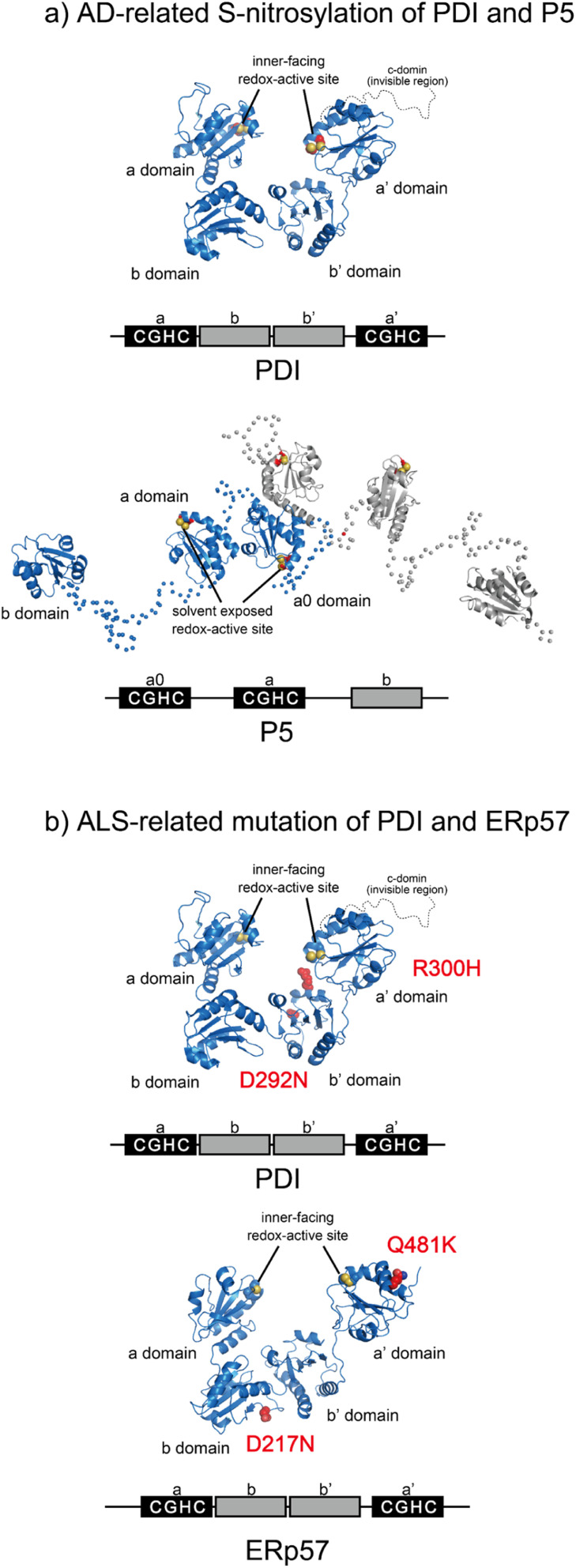
Pathogenic variants of PDI (PDB ID: 4EKZ),^80^ P5, and ERp57 (PDB ID: 6ENY). (a) *S*-Nitrosylation of PDI (left) and P5 (right) has been shown in AD. The Cys of the catalytic sites in each redox active domain are modified by S-NO. (b) ALS-linked variants of PDI and ERp57. ALS variants include D292N and R300H substitutions in the b′ domain of PDI (left). The ERp57 variants D217N and Q481K linked to ALS disrupt neuritogenesis (right).

Most PDIs are multidomain proteins composed of catalytic and noncatalytic thioredoxin (Trx)-like domains.^45,72^ The catalytic domain exhibiting redox activity has a CXXC motif critical for catalyzing thiol–disulfide exchange reactions.^73^ PDIs also exhibit anti-aggregation activity against a broad range of unfolded client proteins.^74–76^ PDIs thus play a key role in maintaining protein homeostasis by not only mediating protein folding *via* catalysis of disulfide bond formation, reduction, and rearrangement but also by chaperoning aggregation-prone clients to decrease the risk of ER stress. Notably, many studies have reported that PDIs play important roles in protein misfolding-related pathologies such as ALS, Alzheimer's disease (AD), Parkinson's disease (PD), and type II diabetes.^15,16,77^ Regarding the relationship between PDIs and diseases related to protein loss-of-function and misfolding, post-translational *S*-nitrosylation of the redox active site in the CXXC motif within PDI and P5 has been shown in AD, indicating possible dysfunction of catalytic oxidative folding (Fig. 4a).^15,78^ ALS risk factor variants include D292N and R300H substitutions in the b′ domain of PDI and D217N and Q481K located in the b and a′ domains of ERp57, respectively (Fig. 4b). These variants disrupt the neurite outgrowth process in zebrafish, leading to motor dysfunction. C57Y, located in the a domain of ERp57, is also a familial intellectual disability risk mutation that causes neurodevelopment defects by disrupting ER proteostasis.^79^ Taken together, extensive biochemical and genetic evidence suggests that PDIs dysfunction lead to pathologies such as neurodegenerative disorders. In this section, we examine the biochemical and structural features of ER-resident PDIs that ensure proper oxidative protein folding fidelity as it is related to pathologic conditions.

### Structural dynamics of PDI engaged in catalysis of oxidative protein folding

The Trx-like domain organization of PDI is designated as an a–b–b′–a′ sequence, forming an overall U-shaped structure (Fig. 4a).^80^ Domains a and a′ contain a redox-active site, whereas domains b and b′ are redox-inactive and function as a principal client binding pocket, especially the b′ domain. An X-linker connects the b′ and a′ domains, and an additional negatively charged α-helical c domain is located in the C-terminus. Regarding the client recognition properties, the redox-dependent client binding and releasing capability of PDI has been reported for several clients, including cholera toxin.^81^ In line with these data, crystallographic analyses demonstrated that the reduced form of PDI has a more closed U-shaped domain arrangement than the oxidized form. A cation–π interaction between the guanidinium group of Arg300 in the b′ domain and the indole ring of Trp396 in the a′ domain contributes to the closed U-shaped structure and is abolished by oxidation of the redox site CXXC in the a′ domain, resulting in an open structure in the oxidized PDI. Accordingly, the inner space within the U-shape in the reduced form is narrower than that in the oxidized form. One interpretation holds that the redox-dependent rearrangement of each domain likely determines the client binding and releasing properties.

**Fig. 5:**
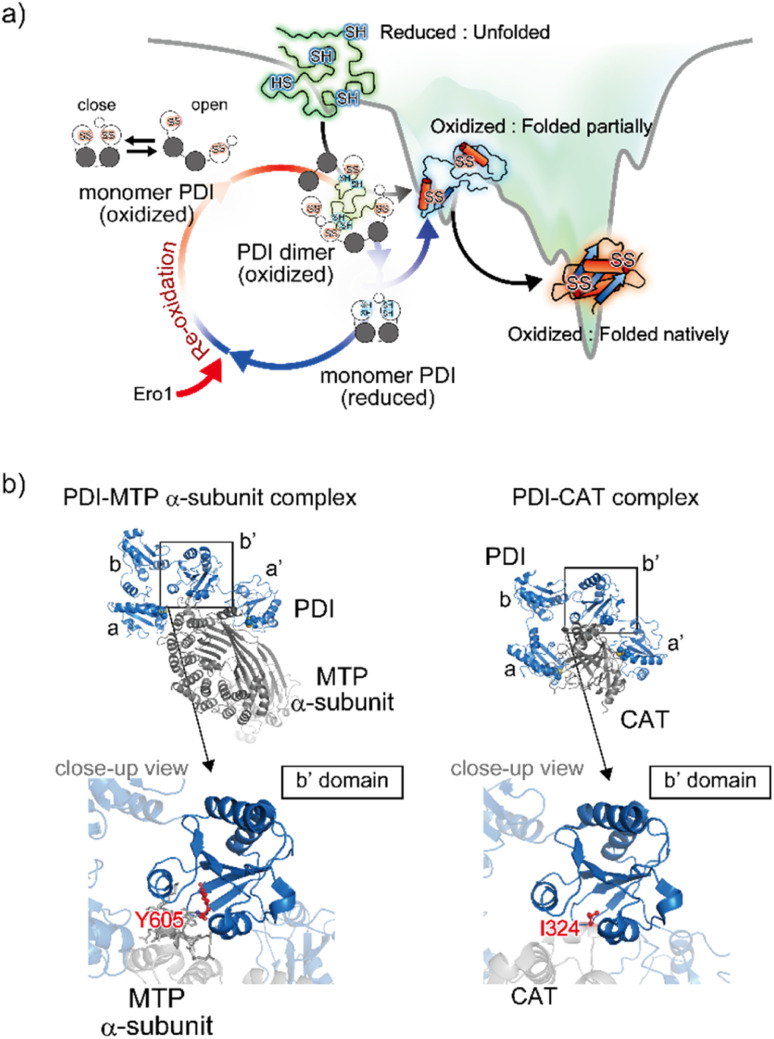
Structural dynamics of PDI engaged in client binding/release. (a) Molecular action of PDI against a client for the catalysis of oxidative folding. The right diagram shows the energy landscape of on-pathway folding to the native conformation *via* a folding intermediate. Along the client folding pathway, oxidized PDI captures an unfolded substrate and assembles to become a face-to-face dimer with a central cavity, inside which the disulfide bonds are introduced into the client. (b) Client binding view based on the complex structures. The left and right panels indicate the complex between PDI and the MTP α-subunit (PDB ID: 6I7S)^85^/CAT (PDB ID: 7ZSC).^86^ Close-up views show the interaction sites between the hydrophobic pocket in the b′ domain of PDI and client-specific residues (Y605 in the MTP α-subunit and I324 in CAT).

Apart from the averaged structure of PDI based on crystallography, single-molecule observations on the time scale of seconds using high-speed atomic force microscopy visualized a rapid equilibrium of oxidized PDI between the open and closed conformations, whereas reduced PDI was maintained in the closed conformation.^82^ Furthermore, sub-millisecond single-molecule observations using single-molecule fluorescence resonance energy transfer showed that the open conformation could be divided into two states.^83^ These two types of single-molecule analysis provided experimental evidence that the intrinsic dynamic nature of PDI switches upon redox.^82,83^ Furthermore, in order to promote the redox-driven cycles of binding and release of client proteins with different shapes and sizes, reduced/denatured bovine pancreatic trypsin inhibitor (BPTI), a model substrate for oxidative folding studies, induced only oxidized PDI to form a transient face-to-face dimer, creating a hydrophobic cavity to accommodate the client protein. Thus, the oxidized form of PDI captures an unstructured client protein, forms face-to-face homodimers that contain multiple redox-active sites and hydrophobic surfaces, and efficiently introduces disulfide bonds into the client protein. Along the folding pathway of the client protein, partially structured folding intermediates are acted on, primarily by monomeric reduced PDI, for proofreading of non-native disulfide bonds of the client protein. Ultimately, correctly folded client proteins with native disulfide bonds are released from PDI (Fig. 5a).

In line with the above interpretation of PDI-catalyzed client folding, regarding the interconvertible populations between the monomeric and dimeric forms, 12%, 26%, and 58% of PDI molecules were observed in the dimeric form in the presence of native, partially folded, and reduced/denatured BPTI, respectively. The dimer with four redox-active sites undergoes repeated expansion and contraction to accelerate the oxidative folding of the client protein inside the cavity. Therefore, the dimeric forms of PDI exhibit conformational transformability and different lifespans that are modulated with respect to the folding status of the individual clients. Given that the size of the client is greater than that of the cavity, multiple dimers can be recruited and function cooperatively to simultaneously promote oxidative folding at multiple sites. Collectively, PDI displays striking redox-dependent conformational dynamics and assembles into dimeric forms to create a transient reaction field along the client folding pathway (Fig. 5a).^84^

In terms of mechanistic insights into client recognition, structural information regarding the complex formed between PDI and the client is limited to that obtained from crystallographic analysis of microsomal triglyceride transfer protein (MTP)/prolyl-4-hydroxylase (P4-H). The α-subunit of MTP, a lipid-binding protein, is aggregation prone, but its aggregation is inhibited by PDI, which recognizes the β-subunit of MTP. Analyses of the MTP complex crystal structure revealed that a protruding loop around the Arg594 to Arg610 domain in the MTP α-subunit interacts with the hydrophobic pocket, namely the principal client-binding site, in the PDI b′ domain (Fig. 5b).^85^ Importantly, Tyr605 of the MTP α-subunit stabilizes the hydrophobic interaction with the PDI b′ domain. In addition to their involvement in client recognition *via* the b′ domain, both the a and a′ domains of PDI contribute to stabilization of the complex. The structure of the complex involving PDI and the C-terminal catalytic (CAT) domain of the P4-H α-subunit further provides important clues for unravelling the client recognition mechanism of PDI. P4-H consists of an α2β2 tetramer and catalyzes the prolyl 4-hydroxylation of procollagen, enabling formation of the triple helical collagen structure. Similar to the MTP α-subunit, the CAT domain interacts with the a, b′, and a′ domains of PDI, also known as the β-subunit of P4-H (Fig. 5b).^86^ Regarding the substrate-binding site in the PDI b′ domain, the side chain of Ile324 in the CAT domain interacts with the hydrophobic pocket of the PDI b′ domain.^85^ Consequently, client recognition by PDI is coordinated in a similar but slightly different fashion depending on the client protein stored in the b′ domain, which is critical for holding the target client.^42,47^

### A unique Leu–Val adhesive motif ensures its function of P5 *via* dimerization

**Fig. 6:**
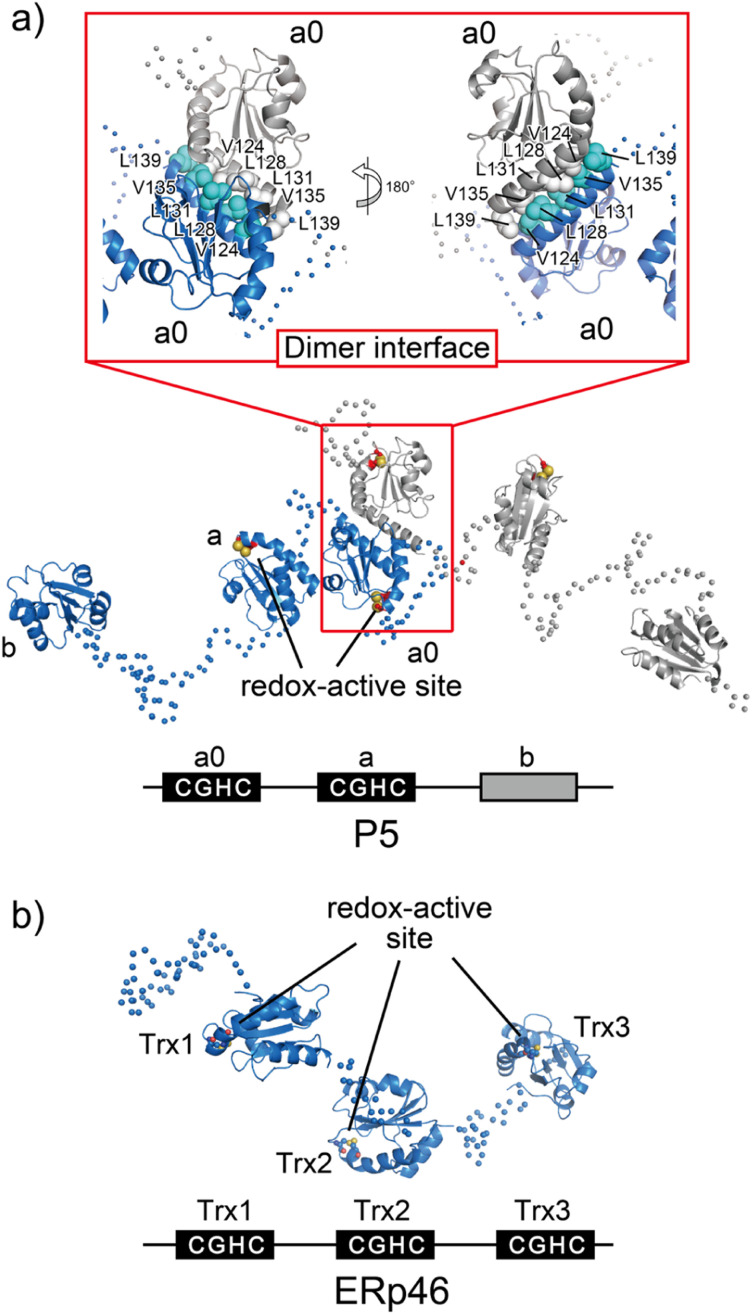
Overall structures of the ER-resident disulfide introducers, P5 and ERp46. (a) P5 dimerizes in solution *via* a unique dimeric motif. The dimeric motif is comprised of a Leu- and Val-rich region in the a_0_ domain, indicated in the inset. Of note, upon mutational impairment of this motif, the protein becomes a monomer in solution that is structurally destabilized around the dimeric interfaces, and monomeric P5 induces ER stress responses in cells. (b) Radically different Trx-like domain arrangement of ERp46. The solvent-exposed redox active sites in each ERp46 Trx-like domain in comparison with PDI work independently. This structural feature is quite different from PDI. The unique domain arrangement of ERp46 with a highly flexible nature among Trx-like domains facilitates the function of ERp46 as an efficient but non-specific disulfide introducer during the catalysis of oxidative folding.

P5, also known as PDIA6, consists of two redox-active Trx-like domains (a_0_ and a) and one redox-inactive Trx-like domain b′ in this order from the N-terminus and dimerizes *via* a unique Leu–Val adhesive motif located in the a′ domain.^74^ Unlike the conventional leucine-zipper motif, in which Leu residues are present every two helical turns of ∼30-residue parallel α-helices, this unique motif consists of a periodic repeat of Leu or Val residues at the third or fourth position spanning five helical turns of the 15-residue anti-parallel α-helices (Fig. 6a). Upon mutational impairment of this motif, P5 becomes a monomer in solution that is structurally destabilized around the dimeric interfaces. When overexpressed in cells, monomeric P5 induces ER stress responses.^74^ Furthermore, the P5 mutant is less capable of inactivating inositol-requiring enzyme 1α, an unfolded response sensor protein, *via* the reduction of intermolecular disulfide bonds. Thus, the unique Leu–Val adhesive motif is critical for stabilizing the overall structure of P5 and facilitating its physiological activity.

As mentioned above, while canonical PDI serves as a versatile catalyst for disulfide-bond formation that slowly but selectively introduces native disulfide bonds into client proteins, P5 and ERp46 catalyze rapid but promiscuous disulfide-bond introduction during the early oxidative folding stages of clients.^87^ PDI and P5/ERp46 presumably catalyze oxidative folding at different stages and function cooperatively to increase the production of secretory and membrane proteins. In line with this mechanism, P5 reportedly interacts with other PDIs^88^ and binding immunoglobulin protein (BiP),^89^ an ER-resident Hsp70-family chaperone. Additionally, a protein–protein interaction analysis showed that PDI and ERp72, as partner proteins of P5, interact non-covalently. P5 then cooperates with PDI to synergistically accelerate the oxidative folding of clients, whereas ERp72-bound P5 up-regulates the chaperone activity.^88^ These data thus provide deeper insights into the protein homeostasis network in the ER, in which the formation of complexes that assist in productive folding is coordinated by several enzymes and chaperones to modulate their enzymatic and chaperone activities.

### Opened V-shape structure of ERp46 as an efficient disulfide bond introducer

ERp46, also known as PDI15 and TXNDC5, contains three redox-active Trx-like domains (Trx1, Trx2, and Trx3) in this order from the N-terminus. These Trx domains are linked by unusually long loops to form a highly flexible open V-shape structure (Fig. 6b).^113^ Studies employing hybrid structural approaches such as small-angle X-ray scattering and crystallographic analyses have investigated this novel domain arrangement and revealed that ERp46 has a radically different structure than PDI. Due to the more solvent-exposed redox-active sites in each ERp46 Trx-like domain compared to PDI, these Trx-like domains seem functionally equivalent. Accordingly, three different ERp46 mutants in which only one Trx-like domain is available for interaction because the other two lack the redox-active sites were shown to introduce disulfide bonds into client proteins at almost the same kinetic rate. This result can be explained by the structural features of ERp46, in which all redox-active sites are contained in the mobile Trx-like domains, which allows easy access to clients by all of the Trx-like domains. Unlike PDI, the unique domain arrangement of ERp46 that renders the Trx-like domains highly flexible is well suited for efficient but non-specific introduction of disulfide bonds during the catalytic reactions of oxidative folding.^113^ Such different domain arrangements among PDIs are common, as they ensure high fidelity for the efficient production of a large quantity of multiple disulfide-bonded proteins. Thus, PDI-catalyzed oxidative folding is a precise but time-consuming process relative to folding catalyzed by ERp46. Hence, PDI and ERp46 synergistically accelerate the overall oxidative folding process, presumably enabling efficient production of folded clients. Regarding the efficient folding catalyzed by ERp46, knockdown of ERp46 in cultured β-cells leads to a significant decrease in insulin production.^90^ Therefore, ERp46 plays a role in β-cell dysfunction and is a therapeutic target in the treatment of diabetes.

## Synthetic regulation of protein folding

### Design of chaperone-mimicking polymeric materials

**Fig. 7:**
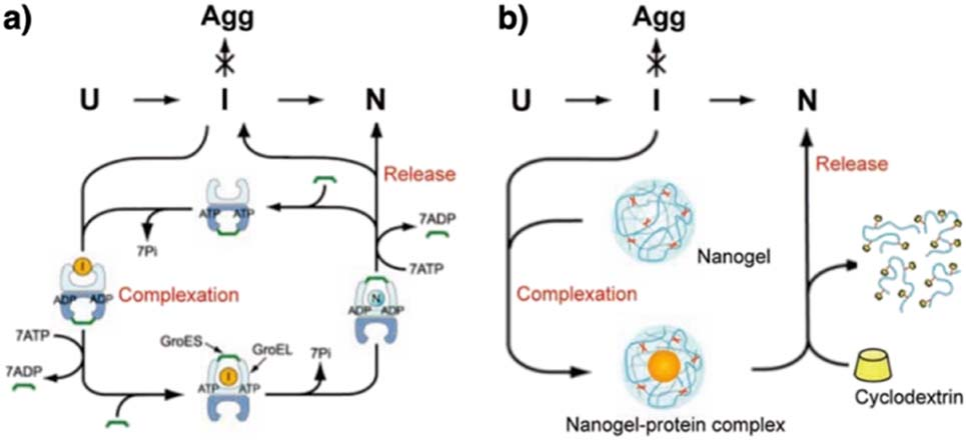
Schematic illustration of (a) chaperone and (b) synthetic nanogel functions that commonly show aggregation inhibition and renaturation of folding intermediates (I). U, Agg, and N denote unfolded, aggregated, and native proteins, respectively. Adapted from ref. 93 with permission from John Wiley and Sons.

The mechanistic details of chaperones and oxidoreductases that catalyze protein folding processes have inspired the design of chaperone- and enzyme-mimetic synthetic aggregation suppressors and folding promoters. Nanogels made of cholesterol-modified polysaccharides represent a pioneering example of chaperone-mimetic synthetic aggregation suppressors (Fig. 7).^91–93^ Cholesteryl group-bearing pullulan (CHP) forms nanogels that interact with proteins primarily through hydrophobic interactions. CHP nanogels can trap denatured proteins produced by heating or chemicals such as urea and guanidium hydrochloride, thereby inhibiting aggregation. By dissociating the nanogels upon complexation with β-cyclodextrin, the trapped proteins can be released for renaturation. Temperature-responsive systems that trap unfolded proteins have also been developed. Co-assembled amphiphilic block copolymers, including poly(*N*-isopropyl acrylamide) (PNIPAM), form core–shell–corona micelles in which the hydrophobic PNIPAM domains are exposed on the surface at temperatures above the lower critical solution temperature (LCST). The hydrophobic domains capture unfolded proteins, thereby inhibiting aggregation. Upon cooling to below the LCST, the PNIPAM domains return to the hydrophilic state, resulting in release of the bound proteins to enable spontaneous folding.^94^ In addition to the amphiphilic macromolecules, ionic graft copolymers show chaperone-like activities. A cationic graft copolymer assembles with an amphiphilic anionic peptide to facilitate the formation of an ordered conformation that inhibits aggregation. The peptide and copolymer assembly shows enhanced bioactivity, such as a membrane disruption function, without loss of water solubility.^95^

### Design of PEG-based chaperone-mimicking molecules

Protein aggregation suppressors based on small molecules are attractive due to the potential advantages of good separation from proteins of interest and little influence on the viscosity of the sample solution. In naturally occurring compounds, arginine and sugars such as cyclodextrins reportedly show aggregation-suppression activity, enabling efficient recovery of the activity of denatured proteins.^96–99^ Using a synthetic approach, researchers used stimuli-responsive building blocks to develop functional molecules capable of suppressing protein aggregation. Poly(ethylene glycol) (PEG) is a water-soluble thermo-responsive macromolecule. The C–C–O repeating unit of PEG, which mostly adopts the *gauche*-form at the C–C bonds, changes conformation upon temperature elevation to increase the ratio of the *anti*-form.^100–105^ Through this *gauche*-to-*anti* conformational change at the C–C bonds, the hydrophobicity of PEG increases, resulting in dehydration. Conventional PEGs dehydrate at quite high temperatures (*i.e.*, >95 °C).^100^ Interestingly, structural modifications resulting in cyclization or amphiphilicity cause PEG to exhibit dehydration at significantly lower temperatures and facilitate functionalities such as protein aggregation suppression.

**Fig. 8:**
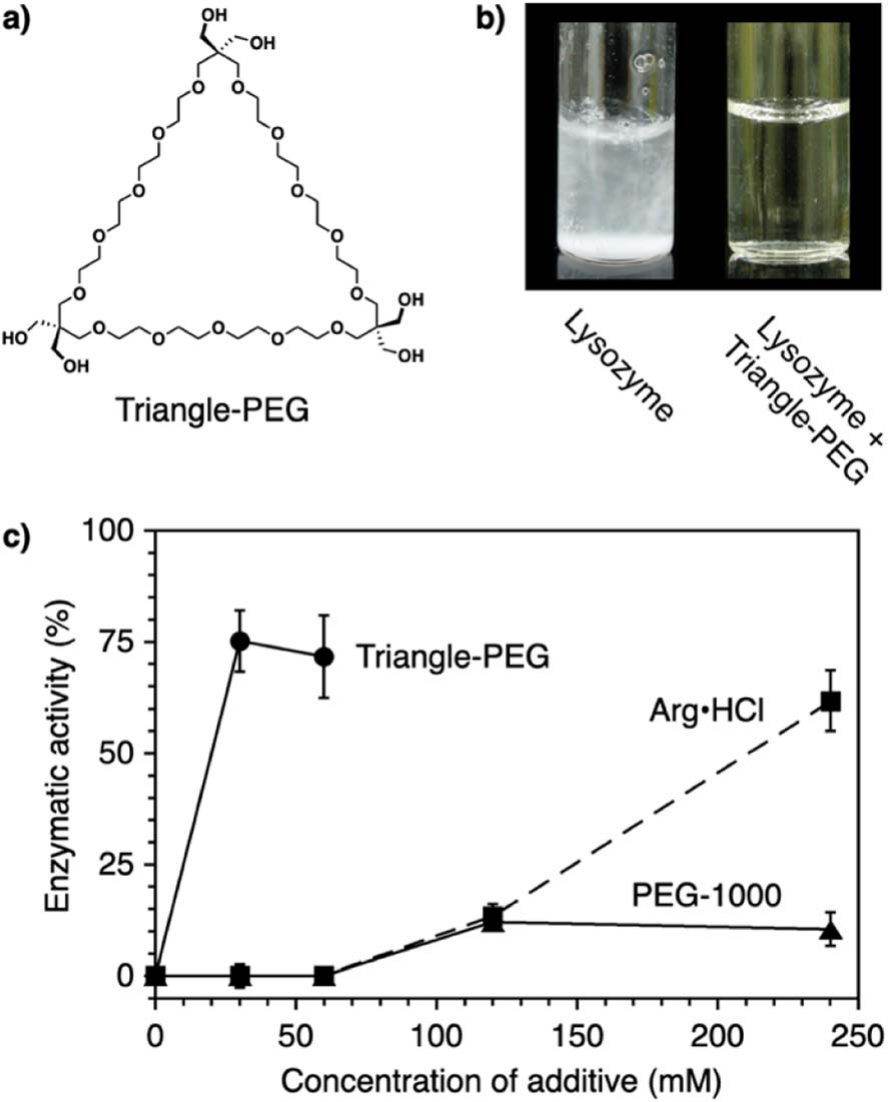
(a) Molecular structure of triangle-PEG. (b) Photographs of lysozyme in PBS at 3.0 mg mL^−1^ (0.21 mM) and 90 °C in the (left) absence and (right) presence of triangle-PEG. (c) Enzymatic activity of lysozyme (3.0 mg mL^−1^) after incubation at 98 °C for 30 min in the presence of triangle-PEG (circles and solid line), PEG-1000 (triangles and solid line), and l-arginine hydrochloride (squares and dashed line).

Triangle-PEG, developed as a cyclized PEG possessing hydroxy groups (Fig. 8a),^132^ consists of three tetraethylene glycol chains connected by pentaerythritols to form a cyclic structure with six hydroxy groups at the corners. ^1^H-NMR relaxation time analyses indicated that triangle-PEG exhibits dehydration at ∼60 °C. The significantly lowered dehydration temperature of triangle-PEG relative to linear PEGs is likely due to a strained geometry caused by cyclization. Triangle-PEG was shown to suppress the aggregation of thermally denatured lysozyme. Lysozyme dissolved in phosphate-buffered saline (PBS) forms aggregates upon heating to 90 °C (Fig. 8b left). In the presence of triangle-PEG, lysozyme in the buffer remained dissolved upon heating (Fig. 8b right). After cooling from 90 °C to 20 °C, circular dichroism (CD) spectroscopy showed recovery of the secondary structure. Aggregation suppression and refolding of thermally denatured lysozyme were evaluated based on enzymatic activity recovery. Even after incubation at 98 °C for 30 min, nearly 80% of the enzymatic activity of lysozyme was recovered in the presence of triangle-PEG (Fig. 8c). In contrast, almost no enzymatic activity was recovered in the presence of linear PEG (PEG-1000). Importantly, triangle-PEG showed more efficient aggregation suppression and refolding of lysozyme than l-arginine and its ester derivative, which have been used as aggregation-suppressing agents in other biological studies.^107–110^ Refolding of the ternary structure of lysozyme was directly monitored by ^1^H-NMR spectroscopy, and fluorescence anisotropy measurements indicated interactions between denatured lysozyme and dehydrated triangle-PEG at high temperatures, leading to suppression of protein aggregation. The hydroxy groups at the vertex of triangle-PEG likely function to afford solubility of the denatured protein by complexation.

**Fig. 9:**
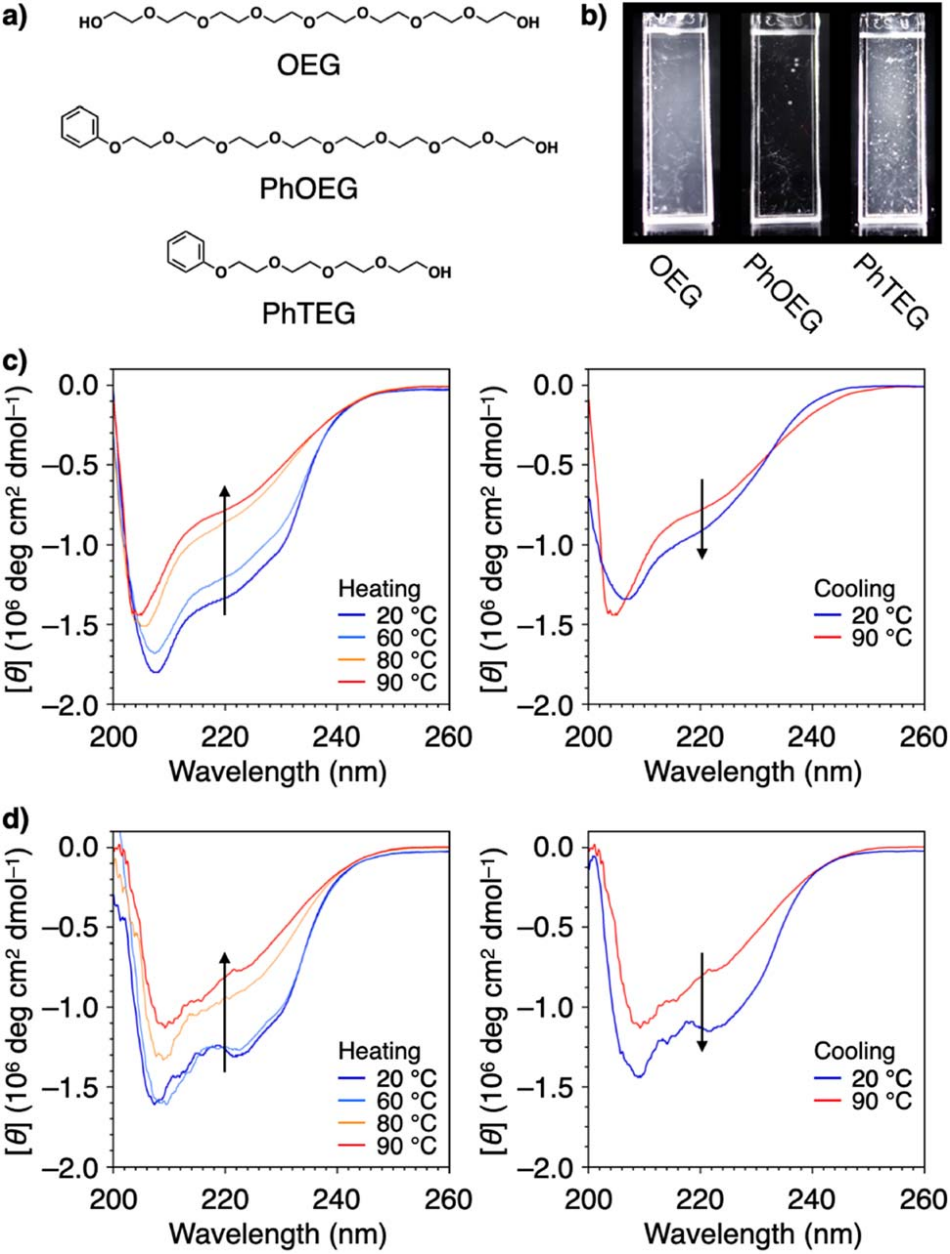
(a) Molecular structures of OEG, PhOEG, and PhTEG. (b) Photographs of lysozyme in PBS at 0.037 mM at 90 °C in the presence of (left) OEG, (center) PhOEG, and (right) PhTEG. CD spectral changes of lysozyme in PBS (0.037 mM) in the presence of (c) OEG (1.5 mM) and (d) PhOEG (1.6 mM) in the (left) heating and (right) cooling processes. Arrows indicate the directions of spectral changes.

Structural modification to impart an amphiphilic nature is another approach used to functionalize PEG for effective protein aggregation suppression. It was reported that PEGs with molecular weights of 2 kDa and 5 kDa with a terminal cholesteryl group stabilize proteins.^111^ Interestingly, even shorter PEGs, such as octaethylene glycol (OEG, 370 Da), acquire the ability to suppress protein aggregation by substitution with a phenyl group at the terminus (PhOEG, Fig. 9a).^112^ Whereas OEG is hydrated over the temperature range of 30–80 °C, PhOEG showed dehydration at approximately 50 °C. The amphiphilic structure is likely responsible for the lowered dehydration temperature. PhOEG exhibiting a thermal response inhibited aggregation of lysozyme upon heating (Fig. 9b). As aggregation suppression was observed above the critical aggregation concentration of PhOEG, it is likely that the self-assembled form of PhOEG functions in protein stabilization. CD spectroscopic analyses showed recovery of the higher-order structures of lysozyme in the presence of PhOEG upon cooling from 90 °C, and the enzymatic activity of lysozyme was recovered at a high yield (78%, Fig. 9c and d) after the cooling process. One plausible mechanism of protein stabilization is that interactions between the hydrophobic surfaces of the denaturing protein and the phenyl appendage of PhOEG afford sufficient water solubility to the denatured protein molecules at high temperatures, thereby enabling their spontaneous refolding in the cooling process. As OEG or PhTEG, a derivative of PhOEG with a tetraethylene glycol chain, exhibited minimal ability to suppress protein aggregation, amphiphilicity plays a critical role in protein stabilization, and OEG is likely the shortest ethylene glycol chain for this function.

### Design of thiol-based oxidoreductase-mimicking molecules

**Fig. 10:**
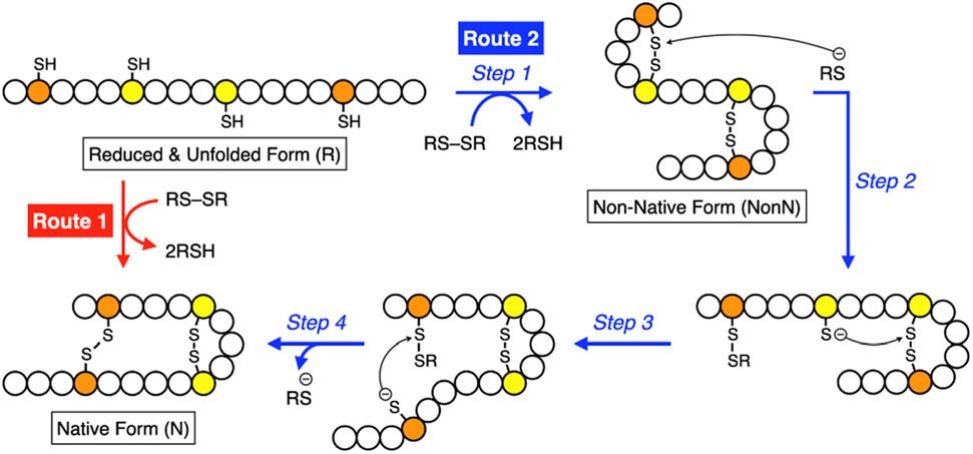
Scheme of oxidative folding of a polypeptide chain forming two disulfide bonds. Orange and yellow circles represent cysteine residues that form disulfide bonds between the same-color pairs, and white circles represent amino acid residues other than cysteine. Route 1: oxidation reaction directly from a reduced and unfolded form (R) to the native form (N). Route 2: oxidation reaction (step 1) followed by disulfide bond isomerization (steps 2–4) from R to N *via* the non-native form (NonN).

Synthetic mimics of oxidoreductases, such as PDI, constitute an important class of disulfide-coupled protein-folding promoters. A reduced and unfolded (R) polypeptide chain bearing multiple cysteine residues would proceed through several folding pathways (Fig. 10). One pathway produces the native form (N) through a straightforward reaction with an oxidant (RS-SR, route 1 in Fig. 10), whereas other routes proceed through the formation of a non-native (NonN) form with disulfide bonds between non-native cysteine pairs (route 2). The subsequent disulfide bond isomerization process enables the conversion of NonN to N, increasing the thermodynamic stability by cleavage and re-formation of disulfide bonds through inter- and intramolecular nucleophilic attacks of thiolate anions (steps 2–4 in route 2). Intracellularly, oxidoreductases assist oxidative protein folding by facilitating the formation and isomerization of disulfide bonds in substrate proteins.^82,84,113^ The Trx-like domains of oxidoreductases such as PDI contain redox-functional active centers with the sequence CXHC.^114^ The thiol group of the N-terminal cysteine residue in the CXHC motif has a lower p*K*_a_ near 7.5,^115^ and the high acidity of the thiol groups efficiently produces thiolate (S^−^) species reactive for disulfide-bond isomerization under physiological conditions *via* nucleophilic attack.

Glutathione (GSH) is a redox-active additive in medium used to promote the folding of denatured proteins. Chemical tuning of GSH modulates its protein-folding activity. For instance, replacement of the glutamic acid residue in GSH with arginine enhances the folding efficiency of reduced and denatured proteins.^39^ In a folding assay of reduced lysozyme, addition of a glutathione derivative modified with arginine showed up to 1.85-times enhanced recovery of the native-structure formation compared to the addition of GSH.

**Fig. 11:**
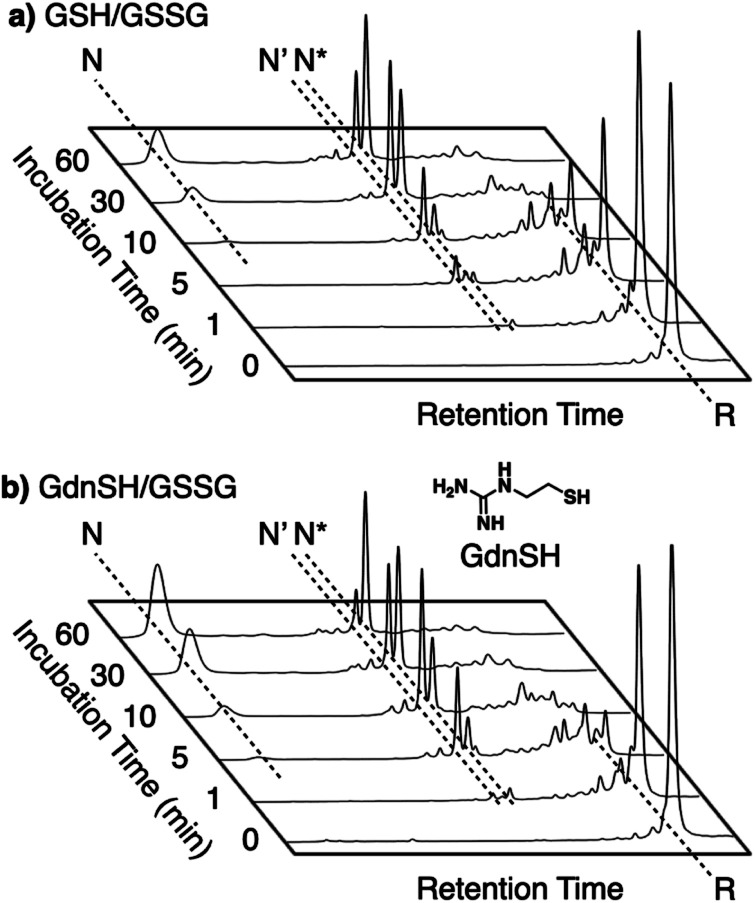
Time-course reverse-phase HPLC analyses of oxidative folding of BPTI (30 μM) in the presence of GSSG (0.20 mM) and (a) GSH or (b) GdnSH (1.0 mM) using water and CH_3_CN containing 0.05% TFA as eluents with a linear gradient at a flow rate of 1.0 mL min^−1^, with absorption monitored at 229 nm and 25 °C.

Enhanced folding efficiencies were also demonstrated by GdnSH, which consists of covalently coupled thiol and guanidyl units.^116^ In the oxidative folding of BPTI with three disulfide bonds, a 60 min incubation in the presence of oxidized glutathione (GSSG) resulted in formation of the native structure at 21% yield ([BPTI] = 30 μM, [thiol] = 1.0 mM, [GSSG] = 0.20 mM). The addition of GSH under these conditions resulted in a slight increase in the refolding yield to 24% (Fig. 11a). Importantly, the GdnSH/GSSG system afforded native BPTI at 51% yield (Fig. 11b). A similar refolding-promotion effect of GdnSH was also observed with ribonuclease (RNase) A. It is likely that the chemical properties of GdnSH and its oxidized form contributed significantly to the folding-promotion effect. Namely, GdnSH/GdnSS has a higher *E*°′ value than GSH/GSSG, indicating that GdnSS exhibits stronger oxidizing power (*E*°′ values of GdnSH/GdnSS: −237 mV *versus* the standard hydrogen electrode, GSH/GSSG: −256 mV, GdnSS: oxidized form of GdnSH). This property of GdnSS allows for more rapid disulfide bond formation of the client protein. In addition, the thiol group of GdnSH is more acidic than that of GSH; therefore, GdnSH should be more nucleophilic (p*K*_a_ values of GdnSH: 8.86, GSH: 9.15). Due to this property, GdnSH likely encourages shuffling of the disulfide bonds to lead to the formation of a thermodynamically stable structure (*i.e.*, the native conformation).

**Fig. 12:**
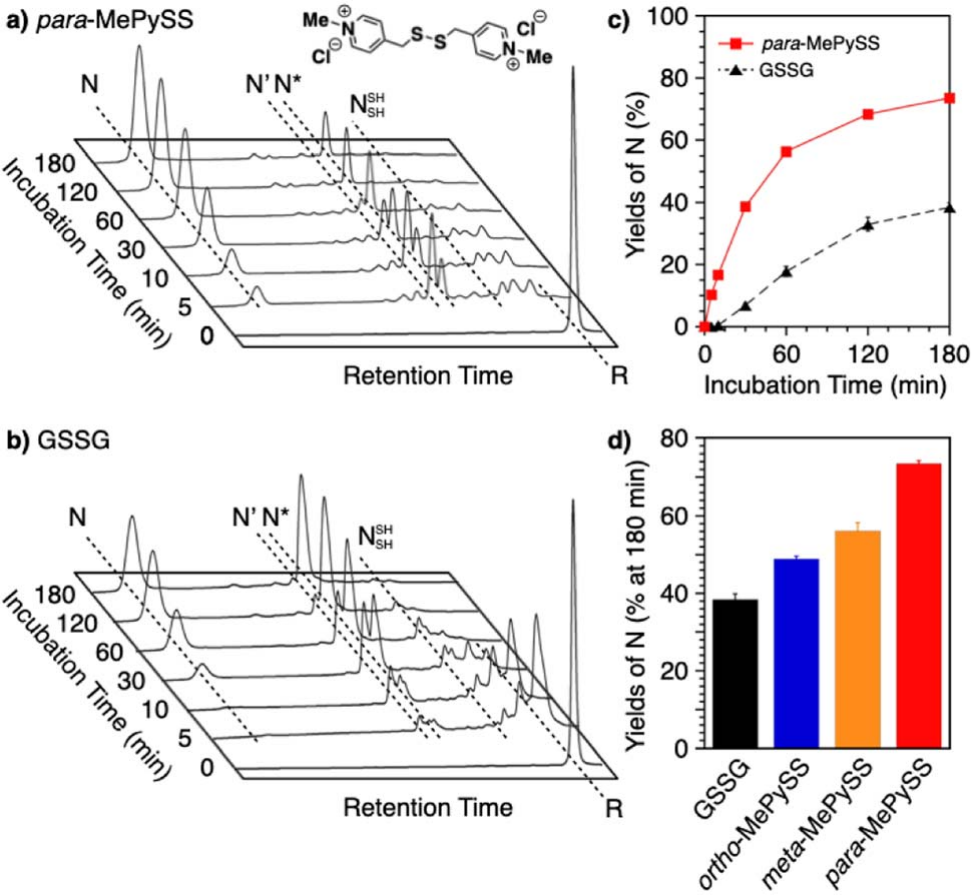
Time-course reverse-phase HPLC analyses of oxidative folding of BPTI (30 μM) in the presence of (a) *para*-MePySS (90 μM) and (b) GSSG (90 μM) using water and CH_3_CN containing 0.05% TFA as eluents with a linear gradient at a flow rate of 1.0 mL min^−1^, with absorption monitored at 229 nm and 30 °C. (c) Time course plots of the yield of N-BPTI in the presence of *para*-MePySS and GSSG (based on a and b). (d) Bar graph indicating the yield of N-BPTI after a 180 min incubation in the presence of GSSG, *ortho*-MePySS, *meta*-MePySS, or *para*-MePySS. The molecular structure of *para*-MePySS is shown in (a). Data are shown as the mean of three independent experiments, with error bars indicating the SEM.

Intracellularly, oxidoreductases bind to unfolded client proteins to catalytically prompt the formation and shuffling of disulfide bonds to induce formation of the native structure. In contrast to such enzymatic reactions, conventional synthetic compounds as protein-folding promoters are commonly used at high concentrations, typically millimolar-order, against substrate proteins on the order of several micromolar concentrations. The development of synthetic protein-folding promoters with activities as high as oxidoreductases showing one-to-one reaction is critically important for lab-scale protein synthesis and industrial production of protein pharmaceuticals. To develop synthetic protein-folding promoters with enhanced activity, thiol and corresponding disulfide compounds exhibiting high nucleophilicity and oxidizability are needed to effectively promote the formation and isomerization of disulfide bonds. To satisfy these properties, methylation of heteroaromatic thiols has been investigated. Methylation is a commonly observed posttranslational modification that regulates the functions of biomacromolecules.^117,118^ Methylation at a heteroatom in the side chain of a protein amino acid alters the electrical properties due to the added cationic charge, which allows for changes in the conformational and functional properties. Chaperones are regulated by methylation; Hsp90, for example, is methylated by a methyltransferase to trigger a conformational change that modulates the interaction with the substrate protein.^119–121^ Inspired by such methylation-associated functional regulation of chaperones, researchers developed heteroaromatic thiols (*i.e.*, *ortho*-, *meta*-, and *para*-substituted pyridinylmethanethiols [PySHs]) as synthetic protein-folding promoters responsive to methylation (Fig. 12a).^38^ It is expected that the basic property of the pyridinyl moiety could increase the acidity of the conjugated thiol group. The pyridinyl moiety is also useful to study the effect of methylation because of its reactivity with halogenated methane. Among the constitutional isomers, *para*-PySHs showed the highest efficiency in terms of protein refolding from the reduced and unfolded states to the native form in the presence of GSSG as an oxidant (yields of native BPTI: 38% by *ortho*-PySH, 48% by *meta*-PySH, 51% by *para*-PySH, [BPTI] = 30 μM, [thiols] = 1.0 mM, [GSSG] = 0.20 mM). Interestingly, *N*-methylation of *para*-PySH significantly enhanced the protein-folding activity (yield of native BPTI: 71% by *para*-MePySH). The enhanced activity of *para*-MePySH is likely due to the stronger oxidizability of its disulfide form and greater nucleophilicity than *para*-PySH, as indicated by the elevated redox potential (*E*°′) and lowered p*K*_a_ value of *para*-MePySH/*para*-MePySS, respectively (*para*-PySH: *E*°′ = −246 mV, p*K*_a_ = 8.68; *para*-MePySH: *E*°′ = −211 mV, p*K*_a_ = 7.34). Based on the high performance of *para*-MePySH, promotion of folding under more biomimetic conditions was investigated. As a semi-enzymatic assay, one equivalent of *para*-MePySS relative to the number of disulfide bonds in the substrate protein was added to the folding medium ([*para*-MePySS] = 90 μM, [reduced BPTI] = 30 μM). Interestingly, this semi-enzymatic reaction condition afforded native BPTI at high yield (74%, Fig. 12a and c). Under identical conditions, the addition of GSSG (90 μM) resulted in the formation of native BPTI at only 38% yield (Fig. 12b), and other constitutional isomers of MePySS showed inferior folding promotion activity against *para*-MePySS (Fig. 12d). The characteristic chemical effect of *N*-methylation of heteroaromatic thiols allowed for marked protein-folding promotion by minimum loading of *para*-MePySH. Such enzyme-mimetic agents could potentially replace dysfunctional enzymes and thereby prevent the formation of pathogenic protein aggregates that cause neurodegenerative diseases and diabetes.

**Fig. 13:**
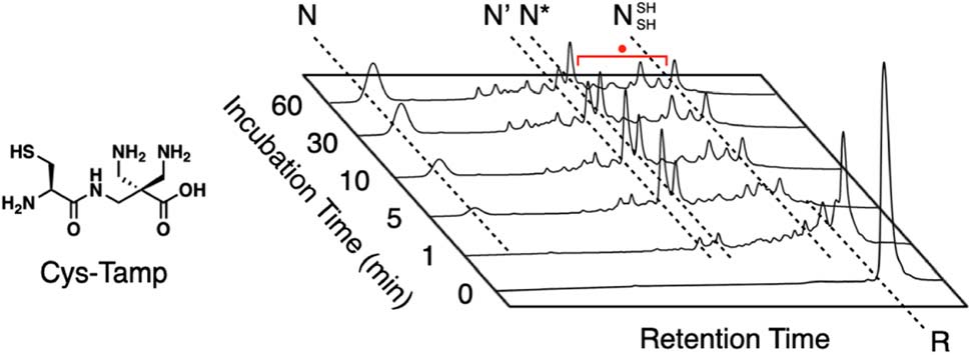
Time-course reversed-phase HPLC analyses of oxidative folding of BPTI (30 μM) in the presence of GSSG (0.20 mM) and Cys-Tamp (1.0 mM) using water and CH_3_CN containing 0.05% TFA as eluents with a linear gradient at a flow rate of 1.0 mL min^−1^, with absorption monitored at 229 nm and 25 °C.

In addition to the chemical tuning of thiol compounds to enhance protein-folding efficiency, the development of compounds that trap folding intermediates and misfolded forms has also attracted scientific attention.^122–126^ The trapping of folding intermediates, particularly off-pathway species, and misfolded forms is important for investigating the folding mechanisms and disease-related biological properties of misfolded proteins.^127–131^ For this purpose, changing the balance between the nucleophilicity and redox potential of a thiol group is effective. Cys-Tamp, a dipeptide composed of cysteine and an amino acid residue with appended diamino groups (Tamp), showed rapid oxidation of the free thiols of unfolded BPTI and RNase A (Fig. 13).^132^ Interestingly, the folding reactions driven by a mixture of Cys-Tamp and GSSG afforded non-native forms of the proteins at significantly increased yields. Cys-Tamp showed a p*K*_a_ of 6.67 and *E*°′ of −281 mV. The increased acidity is suggestive of effective nucleophilicity that would facilitate the cleavage of a protein's disulfide bonds formed between cysteine residues. The lowered redox potential of Cys-Tamp indicates that the oxidized state is favorable. Therefore, intermolecular disulfide bond formation between a protein and Cys-Tamp is likely preferential. Existing methodologies for trapping folding intermediates and misfolded forms are limited to essentially irreversible processes, such as genetic mutations and chemical reactions using iodoacetic acid and maleimide-appending compounds. Owing to the intrinsic reversibility of disulfide bonds in response to electrochemical stimuli, the disulfide bond-based approach demonstrated by Cys-Tamp holds great potential for use in novel reversible methodologies for trapping transient and misfolded forms of proteins by forming intermolecular disulfide bonds and restarting the oxidative folding of the trapped forms through subsequent cleavage of the intermolecular disulfide bonds.

## Conclusions and future outlook

An extensive number of studies have reported a relationship between protein homeostasis-related disulfide chemistry and pathologies such as neurodegenerative disorders. Chaperones and redox-active enzymes are the central players in the proteostasis network, and they may even impact cellular destiny. Particularly, dysfunction of the redox-active enzymes, such as PDI, can cause pathological outcomes.^16^ Therefore, pharmacological strategies to modulate the activity of chaperones and enzymes have important translational value, especially for the promotion of protein folding and inhibition of aggregation. Future translational efforts will offer proof of concept for the therapeutic value of manipulating the folding machinery to treat several protein misfolding-related diseases.

Detailed investigations of the mechanisms of chaperones and enzymes in folding assistance provide fundamentals for genetic and synthetic approaches to enhancing, reinforcing, and emulating the protein activities. For instance, by combining with computational approaches, chaperones and enzymes will be remodeled for desirable properties. Tuning the active center and optimization of the global structure and oligomerization functionality *in silico* should create genetically evolved chaperones and oxidoreductases with enhanced structural robustness and activities. Designing chemical cofactors and modulators will also be advantageous to enhance the activities and reinforce the structural stability. Chemical mimics could also be useful for promoting protein folding and refolding to reduce pathological risks caused by misfolding and aggregation. Currently, the usage of the chaperone and oxidoreductase mimics is limited *in vitro*. For therapeutic studies, it is a critical challenge to expand the environments of the chemical mimics to *in vivo*. Delivery of the mimics into cells and specific organelles should be a primary subject to overcome. Molecular design with membrane permeability and organelle-targeting capabilities will be a promising approach to propel the chemistry of protein folding promotors forward to biological and therapeutic applications. Innovative advances in protein folding chemistry should be achieved by crosscutting research integrating the fundamental mechanistic elucidation of chaperones and redox-active enzymes, synthetic and computational approaches to the development of emulators and engineered proteins, and in-cell applications, enabling the treatment and prevention of protein misfolding-related diseases by recovering proteostasis activities.

## Data availability

There is no original experimental data associated with this article.

## Author contributions

Takahiro Muraoka outlined the first draft and contributed to writing the section “Synthetic regulation of protein folding”. Masaki Okumura contributed to writing the section “ER-resident PDI family members as disulfide-catalysts to ensure oxidative protein folding fidelity”. Tomohide Saio contributed to writing the section “Unraveling the mechanism of protein folding and holding by molecular chaperones”. All authors contributed to writing the sections “Introduction” and “Conclusions and future outlook”.

## Conflicts of interest

There are no conflicts of interest to declare.

## Supplementary Material

## References

[cit1] Kim S. J., Yoon J. S., Shishido H., Yang Z., Rooney L. A., Barral J. M., Skach W. R. (2015). Protein folding. Translational tuning optimizes nascent protein folding in cells. Science.

[cit2] Gething M. J., Sambrook J. (1992). Protein folding in the cell. Nature.

[cit3] Anfinsen C. B. (1973). Principles that govern the folding of protein chains. Science.

[cit4] Dobson C. M. (2003). Protein folding and misfolding. Nature.

[cit5] Sohl J. L., Jaswal S. S., Agard D. A. (1998). Unfolded conformations of alpha-lytic protease are more stable than its native state. Nature.

[cit6] Ptitsyn O. B., Bychkova V. E., Uversky V. N. (1995). Kinetic and equilibrium folding intermediates. Philos. Trans. R. Soc., B.

[cit7] Ren J., Bi Y., Sowers J. R., Hetz C., Zhang Y. (2021). Endoplasmic reticulum stress and unfolded protein response in cardiovascular diseases. Nat. Rev. Cardiol..

[cit8] Lansbury Jr P. T. (1999). Evolution of amyloid: what normal protein folding may tell us about fibrillogenesis and disease. Proc. Natl. Acad. Sci. U. S. A..

[cit9] Fitzpatrick A. W. P., Falcon B., He S., Murzin A. G., Murshudov G., Garringer H. J., Crowther R. A., Ghetti B., Goedert M., Scheres S. H. W. (2017). Cryo-EM structures of tau filaments from Alzheimer's disease. Nature.

[cit10] Lukacs G. L., Verkman A. S. (2012). CFTR: folding, misfolding and correcting the ΔF508 conformational defect. Trends Mol. Med..

[cit11] Ellis J. (1987). Proteins as molecular chaperones. Nature.

[cit12] Kim Y. E., Hipp M. S., Bracher A., Hayer-Hartl M., Hartl F. U. (2013). Molecular chaperone functions in protein folding and proteostasis. Annu. Rev. Biochem..

[cit13] Hipp M. S., Park S. H., Hartl F. U. (2014). Proteostasis impairment in protein-misfolding and -aggregation diseases. Trends Cell Biol..

[cit14] Hipp M. S., Kasturi P., Hartl F. U. (2019). The proteostasis network and its decline in ageing. Nat. Rev. Mol. Cell Biol..

[cit15] Uehara T., Nakamura T., Yao D., Shi Z. Q., Gu Z., Ma Y., Masliah E., Nomura Y., Lipton S. A. (2006). S-nitrosylated protein-disulphide isomerase links protein misfolding to neurodegeneration. Nature.

[cit16] Matsusaki M., Kanemura S., Kinoshita M., Lee Y. H., Inaba K., Okumura M. (2020). The Protein Disulfide Isomerase Family: from proteostasis to pathogenesis. Biochim. Biophys. Acta, Gen. Subj..

[cit17] Balchin D., Hayer-Hartl M., Hartl F. U. (2016). vivo aspects of protein folding and quality control. Science.

[cit18] Fiedorczuk K., Chen J. (2022). Mechanism of CFTR correction by type I folding correctors. Cell.

[cit19] Zimmerman S. B., Trach S. O. (1991). Estimation of macromolecule concentrations and excluded volume effects for the cytoplasm of Escherichia coli. J. Mol. Biol..

[cit20] Hartl F. U., Bracher A., Hayer-Hartl M. (2011). Molecular chaperones in protein folding and proteostasis. Nature.

[cit21] Tian G., Vainberg I. E., Tap W. D., Lewis S. A., Cowan N. J. (1995). Specificity in chaperonin-mediated protein folding. Nature.

[cit22] Scholz C., Stoller G., Zarnt T., Fischer G., Schmid F. X. (1997). Cooperation of enzymatic and chaperone functions of trigger factor in the catalysis of protein folding. EMBO J..

[cit23] Hardy S. J., Randall L. L. (1991). A kinetic partitioning model of selective binding of nonnative proteins by the bacterial chaperone SecB. Science.

[cit24] Morimoto R. I., Kline M. P., Bimston D. N., Cotto J. J. (1997). The heat-shock response: regulation and function of heat-shock proteins and molecular chaperones. Essays Biochem..

[cit25] Boisvert D. C., Wang J., Otwinowski Z., Horwich A. L., Sigler P. B. (1996). The 2.4 A crystal structure of the bacterial chaperonin GroEL complexed with ATP gamma S. Nat. Struct. Biol..

[cit26] Liu Z., Zhang S., Gu J., Tong Y., Li Y., Gui X., Long H., Wang C., Zhao C., Lu J., He L., Li Y., Liu Z., Li D., Liu C. (2020). Hsp27 chaperones FUS phase separation under the modulation of stress-induced phosphorylation. Nat. Struct. Mol. Biol..

[cit27] Gu J., Liu Z., Zhang S., Li Y., Xia W., Wang C., Xiang H., Liu Z., Tan L., Fang Y., Liu C., Li D. (2020). Hsp40 proteins phase separate to chaperone the assembly and maintenance of membraneless organelles. Proc. Natl. Acad. Sci. U. S. A..

[cit28] Yoshizawa T., Ali R., Jiou J., Fung H. Y. J., Burke K. A., Kim S. J., Lin Y., Peeples W. B., Saltzberg D., Soniat M., Baumhardt J. M., Oldenbourg R., Sali A., Fawzi N. L., Rosen M. K., Chook Y. M. (2018). Nuclear Import Receptor Inhibits Phase Separation of FUS through Binding to Multiple Sites. Cell.

[cit29] Nanaura H., Kawamukai H., Fujiwara A., Uehara T., Aiba Y., Nakanishi M., Shiota T., Hibino M., Wiriyasermkul P., Kikuchi S., Nagata R., Matsubayashi M., Shinkai Y., Niwa T., Mannen T., Morikawa N., Iguchi N., Kiriyama T., Morishima K., Inoue R., Sugiyama M., Oda T., Kodera N., Toma-Fukai S., Sato M., Taguchi H., Nagamori S., Shoji O., Ishimori K., Matsumura H., Sugie K., Saio T., Yoshizawa T., Mori E. (2021). C9orf72-derived arginine-rich poly-dipeptides impede phase modifiers. Nat. Commun..

[cit30] Frottin F., Schueder F., Tiwary S., Gupta R., Körner R., Schlichthaerle T., Cox J., Jungmann R., Hartl F. U., Hipp M. S. (2019). The nucleolus functions as a phase-separated protein quality control compartment. Science.

[cit31] Braakman I., Bulleid N. J. (2011). Protein folding and modification in the mammalian endoplasmic reticulum. Annu. Rev. Biochem..

[cit32] Arolas J. L., Aviles F. X., Chang J. Y., Ventura S. (2006). Folding of small disulfide-rich proteins: clarifying the puzzle. Trends Biochem. Sci..

[cit33] Okumura M., Shimamoto S., Hidaka Y. (2012). A chemical method for investigating disulfide-coupled peptide and protein folding. FEBS J..

[cit34] Weissman J. S., Kim P. S. (1995). A kinetic explanation for the rearrangement pathway of BPTI folding. Nat. Struct. Biol..

[cit35] Weissman J. S., Kim P. S. (1991). Reexamination of the folding of BPTI: predominance of native intermediates. Science.

[cit36] Creighton T. E. (1974). Intermediates in the refolding of reduced pancreatic trypsin inhibitor. J. Mol. Biol..

[cit37] Redfield C., Schulman B. A., Milhollen M. A., Kim P. S., Dobson C. M. (1999). Alpha-lactalbumin forms a compact molten globule in the absence of disulfide bonds. Nat. Struct. Biol..

[cit38] Okada S., Matsumoto Y., Takahashi R., Arai K., Kanemura S., Okumura M., Muraoka T. (2023). Semi-enzymatic acceleration of oxidative protein folding by N-methylated heteroaromatic thiols. Chem. Sci..

[cit39] Okumura M., Saiki M., Yamaguchi H., Hidaka Y. (2011). Acceleration of disulfide-coupled protein folding using glutathione derivatives. FEBS J..

[cit40] Huth J. R., Perini F., Lockridge O., Bedows E., Ruddon R. W. (1993). Protein folding and assembly in vitro parallel intracellular folding and assembly. Catalysis of folding and assembly of the human chorionic gonadotropin alpha beta dimer by protein disulfide isomerase. J. Biol. Chem..

[cit41] Tamura T., Arai S., Nagaya H., Mizuguchi J., Wada I. (2013). Stepwise assembly of fibrinogen is assisted by the endoplasmic reticulum lectin-chaperone system in HepG2 cells. PLoS One.

[cit42] Kanemura S., Matsusaki M., Inaba K., Okumura M. (2020). PDI Family Members as Guides for Client Folding and Assembly. Int. J. Mol. Sci..

[cit43] Weissman J. S., Kim P. S. (1993). Efficient catalysis of disulphide bond rearrangements by protein disulphide isomerase. Nature.

[cit44] van den Berg B., Chung E. W., Robinson C. V., Mateo P. L., Dobson C. M. (1999). The oxidative refolding of hen lysozyme and its catalysis by protein disulfide isomerase. EMBO J..

[cit45] Okumura M., Kadokura H., Inaba K. (2015). Structures and functions of protein disulfide isomerase family members involved in proteostasis in the endoplasmic reticulum. Free Radical Biol. Med..

[cit46] Lumb R. A., Bulleid N. J. (2002). Is protein disulfide isomerase a redox-dependent molecular chaperone?. EMBO J..

[cit47] Okumura M., Kadokura H., Hashimoto S., Yutani K., Kanemura S., Hikima T., Hidaka Y., Ito L., Shiba K., Masui S., Imai D., Imaoka S., Yamaguchi H., Inaba K. (2014). Inhibition of the functional interplay between endoplasmic reticulum (ER) oxidoreduclin-1α (Ero1α) and protein-disulfide isomerase (PDI) by the endocrine disruptor bisphenol A. J. Biol. Chem..

[cit48] Matsuda J., Suzuki O., Oshima A., Yamamoto Y., Noguchi A., Takimoto K., Itoh M., Matsuzaki Y., Yasuda Y., Ogawa S., Sakata Y., Nanba E., Higaki K., Ogawa Y., Tominaga L., Ohno K., Iwasaki H., Watanabe H., Brady R. O., Suzuki Y. (2003). Chemical chaperone therapy for brain pathology in G(M1)-gangliosidosis. Proc. Natl. Acad. Sci. U. S. A..

[cit49] Hartl F. U. (2017). Protein Misfolding Diseases. Annu. Rev. Biochem..

[cit50] Bascos N. A. D., Landry S. J. (2019). A History of Molecular Chaperone Structures in the Protein Data Bank. Int. J. Mol. Sci..

[cit51] Xu Z., Horwich A. L., Sigler P. B. (1997). The crystal structure of the asymmetric GroEL–GroES–(ADP)7 chaperonin complex. Nature.

[cit52] Cong Y., Schröder G. F., Meyer A. S., Jakana J., Ma B., Dougherty M. T., Schmid M. F., Reissmann S., Levitt M., Ludtke S. L., Frydman J., Chiu W. (2012). Symmetry-free cryo-EM structures of the chaperonin TRiC along its ATPase-driven conformational cycle. EMBO J..

[cit53] Shiau A. K., Harris S. F., Southworth D. R., Agard D. A. (2006). Structural Analysis of E. coli hsp90 reveals dramatic nucleotide-dependent conformational rearrangements. Cell.

[cit54] Ali M. M., Roe S. M., Vaughan C. K., Meyer P., Panaretou B., Piper P. W., Prodromou C., Pearl L. H. (2006). Crystal structure of an Hsp90-nucleotide-p23/Sba1 closed chaperone complex. Nature.

[cit55] Saio T., Guan X., Rossi P., Economou A., Kalodimos C. G. (2014). Structural basis for protein antiaggregation activity of the trigger factor chaperone. Science.

[cit56] Tugarinov V., Kanelis V., Kay L. E. (2006). Isotope labeling strategies for the study of high-molecular-weight proteins by solution NMR spectroscopy. Nat. Protoc..

[cit57] Kainosho M., Torizawa T., Iwashita Y., Terauchi T., Mei Ono A., Güntert P. (2006). Optimal isotope labelling for NMR protein structure determinations. Nature.

[cit58] Schütz S., Sprangers R. (2020). Methyl TROSY spectroscopy: A versatile NMR approach to study challenging biological systems. Prog. Nucl. Magn. Reson. Spectrosc..

[cit59] Saio T., Kawagoe S., Ishimori K., Kalodimos C. G. (2018). Oligomerization of a molecular chaperone modulates its activity. eLife.

[cit60] Mas G., Guan J. Y., Crublet E., Debled E. C., Moriscot C., Gans P., Schoehn G., Macek P., Schanda P., Boisbouvier J. (2018). Structural investigation of a chaperonin in action reveals how nucleotide binding regulates the functional cycle. Sci. Adv..

[cit61] Huang C., Rossi P., Saio T., Kalodimos C. G. (2016). Structural basis for the antifolding activity of a molecular chaperone. Nature.

[cit62] Jiang Y., Rossi P., Kalodimos C. G. (2019). Structural basis for client recognition and activity of Hsp40 chaperones. Science.

[cit63] Kawagoe S., Ishimori K., Saio T. (2022). Structural and Kinetic Views of Molecular Chaperones in Multidomain Protein Folding. Int. J. Mol. Sci..

[cit64] Ferbitz L., Maier T., Patzelt H., Bukau B., Deuerling E., Ban N. (2004). Trigger factor in complex with the ribosome forms a molecular cradle for nascent proteins. Nature.

[cit65] Dekker C., de Kruijff B., Gros P. (2003). Crystal structure of SecB from Escherichia coli. J. Struct. Biol..

[cit66] Zhu H., Matsusaki M., Sugawara T., Ishimori K., Saio T. (2021). Zinc-Dependent Oligomerization of Thermus thermophilus
Trigger Factor Chaperone. Biology.

[cit67] Suno R., Taguchi H., Masui R., Odaka M., Yoshida M. (2004). Trigger factor from Thermus thermophilus is a Zn2+-dependent chaperone. J. Biol. Chem..

[cit68] Mas G., Burmann B. M., Sharpe T., Claudi B., Bumann D., Hiller S. (2020). Regulation of chaperone function by coupled folding and oligomerization. Sci. Adv..

[cit69] Giese K. C., Vierling E. (2002). Changes in oligomerization are essential for the chaperone activity of a small heat shock protein in vivo and in vitro. J. Biol. Chem..

[cit70] Månsson C., van Cruchten R. T. P., Weininger U., Yang X., Cukalevski R., Arosio P., Dobson C. M., Knowles T., Akke M., Linse S., Emanuelsson C. (2018). Conserved S/T Residues of the Human Chaperone DNAJB6 Are Required for Effective Inhibition of Aβ42 Amyloid Fibril Formation. Biochemistry.

[cit71] Cawood E. E., Clore G. M., Karamanos T. K. (2022). Microsecond Backbone Motions Modulate the Oligomerization of the DNAJB6 Chaperone. Angew. Chem., Int. Ed. Engl..

[cit72] Fass D., Thorpe C. (2018). Chemistry and Enzymology of Disulfide Cross-Linking in Proteins. Chem. Rev..

[cit73] Araki K., Iemura S., Kamiya Y., Ron D., Kato K., Natsume T., Nagata K. (2013). Ero1-α and PDIs constitute a hierarchical electron transfer network of endoplasmic reticulum oxidoreductases. J. Cell Biol..

[cit74] Okumura M., Kanemura S., Matsusaki M., Kinoshita M., Saio T., Ito D., Hirayama C., Kumeta H., Watabe M., Amagai Y., Lee Y. H., Akiyama S., Inaba K. (2021). A unique leucine-valine adhesive motif supports structure and function of protein disulfide isomerase P5 via dimerization. Structure.

[cit75] Tanikawa Y., Kanemura S., Ito D., Lin Y., Matsusaki M., Kuroki K., Yamaguchi H., Maenaka K., Lee Y. H., Inaba K., Okumura M. (2021). Ca(2+) Regulates ERp57-Calnexin Complex Formation. Molecules.

[cit76] Mideksa Y. G., Aschenbrenner I., Fux A., Kaylani D., Weiß C. A. M., Nguyen T. A., Bach N. C., Lang K., Sieber S. A., Feige M. J. (2022). A comprehensive set of ER protein disulfide isomerase family members supports the biogenesis of proinflammatory interleukin 12 family cytokines. J. Biol. Chem..

[cit77] Woehlbier U., Colombo A., Saaranen M. J., Pérez V., Ojeda J., Bustos F. J., Andreu C. I., Torres M., Valenzuela V., Medinas D. B., Rozas P., Vidal R. L., Lopez-Gonzalez R., Salameh J., Fernandez-Collemann S., Muñoz N., Matus S., Armisen R., Sagredo A., Palma K., Irrazabal T., Almeida S., Gonzalez-Perez P., Campero M., Gao F. B., Henny P., van Zundert B., Ruddock L. W., Concha M. L., Henriquez J. P., Brown R. H., Hetz C. (2016). ALS-linked protein disulfide isomerase variants cause motor dysfunction. EMBO J..

[cit78] Honjo Y., Horibe T., Torisawa A., Ito H., Nakanishi A., Mori H., Komiya T., Takahashi R., Kawakami K. (2014). Protein disulfide isomerase P5-immunopositive inclusions in patients with Alzheimer's disease. J. Alzheimer's Dis..

[cit79] Bilches Medinas D., Malik S., Yıldız-Bölükbaşı E., Borgonovo J., Saaranen M. J., Urra H., Pulgar E., Afzal M., Contreras D., Wright M. T., Bodaleo F., Quiroz G., Rozas P., Mumtaz S., Díaz R., Rozas C., Cabral-Miranda F., Piña R., Valenzuela V., Uyan O., Reardon C., Woehlbier U., Brown R. H., Sena-Esteves M., Gonzalez-Billault C., Morales B., Plate L., Ruddock L. W., Concha M. L., Hetz C., Tolun A. (2022). Mutation in protein disulfide isomerase A3 causes neurodevelopmental defects by disturbing endoplasmic reticulum proteostasis. EMBO J..

[cit80] Wang C., Li W., Ren J., Fang J., Ke H., Gong W., Feng W., Wang C. C. (2013). Structural insights into the redox-regulated dynamic conformations of human protein disulfide isomerase. Antioxid. Redox Signaling.

[cit81] Tsai B., Rodighiero C., Lencer W. I., Rapoport T. A. (2001). Protein disulfide isomerase acts as a redox-dependent chaperone to unfold cholera toxin. Cell.

[cit82] Okumura M., Noi K., Kanemura S., Kinoshita M., Saio T., Inoue Y., Hikima T., Akiyama S., Ogura T., Inaba K. (2019). Dynamic assembly of protein disulfide isomerase in catalysis of oxidative folding. Nat. Chem. Biol..

[cit83] Chinnaraj M., Flaumenhaft R., Pozzi N. (2022). Reduction of protein disulfide isomerase results in open conformations and stimulates dynamic exchange between structural ensembles. J. Biol. Chem..

[cit84] Okumura M., Noi K., Inaba K. (2021). Visualization of structural dynamics of protein disulfide isomerase enzymes in catalysis of oxidative folding and reductive unfolding. Curr. Opin. Struct. Biol..

[cit85] Biterova E. I., Isupov M. N., Keegan R. M., Lebedev A. A., Sohail A. A., Liaqat I., Alanen H. I., Ruddock L. W. (2019). The crystal structure of human microsomal triglyceride transfer protein. Proc. Natl. Acad. Sci. U. S. A..

[cit86] Murthy A. V., Sulu R., Lebedev A., Salo A. M., Korhonen K., Venkatesan R., Tu H., Bergmann U., Jänis J., Laitaoja M., Ruddock L. W., Myllyharju J., Koski M. K., Wierenga R. K. (2022). Crystal structure of the collagen prolyl 4-hydroxylase (C-P4H) catalytic domain complexed with PDI: Toward a model of the C-P4H α(2)β(2) tetramer. J. Biol. Chem..

[cit87] Sato Y., Kojima R., Okumura M., Hagiwara M., Masui S., Maegawa K., Saiki M., Horibe T., Suzuki M., Inaba K. (2013). Synergistic cooperation of PDI family members in peroxiredoxin 4-driven oxidative protein folding. Sci. Rep..

[cit88] Matsusaki M., Okada R., Tanikawa Y., Kanemura S., Ito D., Lin Y., Watabe M., Yamaguchi H., Saio T., Lee Y. H., Inaba K., Okumura M. (2021). Functional Interplay between P5 and PDI/ERp72 to Drive Protein Folding. Biology.

[cit89] Jessop C. E., Watkins R. H., Simmons J. J., Tasab M., Bulleid N. J. (2009). Protein disulphide isomerase family members show distinct substrate specificity: P5 is targeted to BiP client proteins. J. Cell Sci..

[cit90] Alberti A., Karamessinis P., Peroulis M., Kypreou K., Kavvadas P., Pagakis S., Politis P. K., Charonis A. (2009). ERp46 is reduced by high glucose and regulates insulin content in pancreatic beta-cells. Am. J. Physiol.: Endocrinol. Metab..

[cit91] Akiyoshi K., Sasaki Y., Sunamoto J. (1999). Molecular chaperone-like activity of hydrogel nanoparticles of hydrophobized pullulan: thermal stabilization with refolding of carbonic anhydrase B. Bioconjugate Chem..

[cit92] Nomura Y., Sasaki Y., Takagi M., Narita T., Aoyama Y., Akiyoshi K. (2005). Thermoresponsive controlled association of protein with a dynamic nanogel of hydrophobized polysaccharide and cyclodextrin: heat shock protein-like activity of artificial molecular chaperone. Biomacromolecules.

[cit93] Sasaki Y., Akiyoshi K. (2010). Nanogel engineering for new nanobiomaterials: from chaperoning engineering to biomedical applications. Chem. Rec..

[cit94] Liu X., Liu Y., Zhang Z., Huang F., Tao Q., Ma R., An Y., Shi L. (2013). Temperature-responsive mixed-shell polymeric micelles for the refolding of thermally denatured proteins. Chemistry.

[cit95] Shimada N., Kinoshita H., Tokunaga S., Umegae T., Kume N., Sakamoto W., Maruyama A. (2015). Inter-polyelectrolyte nano-assembly induces folding and activation of functional peptides. J. Controlled Release.

[cit96] Buchner J., Rudolph R. (1991). Renaturation, purification and characterization of recombinant Fab-fragments produced in Escherichia coli. Bio/Technology.

[cit97] Karuppiah N., Sharma A. (1995). Cyclodextrins as protein folding aids. Biochem. Biophys. Res. Commun..

[cit98] Tsumoto K., Ejima D., Kita Y., Arakawa T. (2005). Review: Why is arginine effective in suppressing aggregation?. Protein Pept. Lett..

[cit99] Hamada H., Arakawa T., Shiraki K. (2009). Effect of additives on protein aggregation. Curr. Pharm. Biotechnol..

[cit100] Hey M. J., Ilett S. M., Davidson G. (1995). Effect of temperature on poly(ethylene oxide) chains in aqueous solution. A viscometric, 1H NMR and Raman spectroscopic study. J. Chem. Soc., Faraday Trans..

[cit101] Bjoerling M., Karlstroem G., Linse P. (1991). Conformational adaption of poly(ethylene oxide): A carbon-13 NMR study. J. Phys. Chem..

[cit102] Matsuura H., Fukuhara K. (1985). Conformational analysis of poly(oxyethylene) chain in aqueous solution as a hydrophilic moiety of nonionic surfactants. J. Mol. Struct..

[cit103] Karlstroem G. (1985). A new model for upper and lower critical solution temperatures in poly(ethylene oxide) solutions. J. Phys. Chem..

[cit104] Saeki S., Kuwahara N., Nakata M., Kaneko M. (1976). Upper and lower critical solution temperatures in poly (ethylene glycol) solutions. Polymer.

[cit105] Matsuura H., Miyazawa T. (1969). Vibrational analysis of molten poly(ethylene glycol). J. Polym. Sci., Part A-2: Polym. Phys..

[cit106] Muraoka T., Adachi K., Ui M., Kawasaki S., Sadhukhan N., Obara H., Tochio H., Shirakawa M., Kinbara K. (2013). A structured monodisperse PEG for the effective suppression of protein aggregation. Angew. Chem., Int. Ed..

[cit107] Lange C., Rudolph R. (2009). Suppression of protein aggregation by L-arginine. Curr. Pharm. Biotechnol..

[cit108] Arakawa T., Ejima D., Tsumoto K., Obeyama N., Tanaka Y., Kita Y., Timasheff S. N. (2007). Suppression of protein interactions by arginine: a proposed mechanism of the arginine effects. Biophys. Chem..

[cit109] Shiraki K., Kudou M., Nishikori S., Kitagawa H., Imanaka T., Takagi M. (2004). Arginine ethylester prevents thermal inactivation and aggregation of lysozyme. Eur. J. Biochem..

[cit110] Arakawa T., Tsumoto K. (2003). The effects of arginine on refolding of aggregated proteins: not facilitate refolding, but suppress aggregation. Biochem. Biophys. Res. Commun..

[cit111] Mueller C., Capelle M. A., Seyrek E., Martel S., Carrupt P. A., Arvinte T., Borchard G. (2012). Noncovalent PEGylation: different effects of dansyl-, L-tryptophan-, phenylbutylamino-, benzyl- and cholesteryl-PEGs on the aggregation of salmon calcitonin and lysozyme. J. Pharm. Sci..

[cit112] Sadhukhan N., Muraoka T., Ui M., Nagatoishi S., Tsumoto K., Kinbara K. (2015). Protein stabilization by an amphiphilic short monodisperse oligo(ethylene glycol). Chem. Commun..

[cit113] Kojima R., Okumura M., Masui S., Kanemura S., Inoue M., Saiki M., Yamaguchi H., Hikima T., Suzuki M., Akiyama S., Inaba K. (2014). Radically different thioredoxin domain arrangement of ERp46, an efficient disulfide bond introducer of the mammalian PDI family. Structure.

[cit114] Edman J. C., Ellis L., Blacher R. W., Roth R. A., Rutter W. J. (1985). Sequence of protein disulphide isomerase and implications of its relationship to thioredoxin. Nature.

[cit115] Chivers P. T., Prehoda K. E., Volkman B. F., Kim B. M., Markley J. L., Raines R. T. (1997). Microscopic pKa values of Escherichia coli thioredoxin. Biochemistry.

[cit116] Okada S., Matsusaki M., Arai K., Hidaka Y., Inaba K., Okumura M., Muraoka T. (2019). Coupling effects of thiol and urea-type groups for promotion of oxidative protein folding. Chem. Commun..

[cit117] Aletta J. M., Cimato T. R., Ettinger M. J. (1998). Protein methylation: a signal event in post-translational modification. Trends Biochem. Sci..

[cit118] Walsh C. T., Garneau-Tsodikova S., Gatto Jr G. J. (2005). Protein posttranslational modifications: the chemistry of proteome diversifications. Angew. Chem., Int. ed. Engl..

[cit119] Rehn A., Lawatscheck J., Jokisch M. L., Mader S. L., Luo Q., Tippel F., Blank B., Richter K., Lang K., Kaila V. R. I., Buchner J. (2020). A methylated lysine is a switch point for conformational communication in the chaperone Hsp90. Nat. Commun..

[cit120] Donlin L. T., Andresen C., Just S., Rudensky E., Pappas C. T., Kruger M., Jacobs E. Y., Unger A., Zieseniss A., Dobenecker M. W., Voelkel T., Chait B. T., Gregorio C. C., Rottbauer W., Tarakhovsky A., Linke W. A. (2012). Smyd2 controls cytoplasmic lysine methylation of Hsp90 and myofilament organization. Genes Dev..

[cit121] Abu-Farha M., Lanouette S., Elisma F., Tremblay V., Butson J., Figeys D., Couture J. F. (2011). Proteomic analyses of the SMYD family interactomes identify HSP90 as a novel target for SMYD2. J. Mol. Cell Biol..

[cit122] Neudecker P., Robustelli P., Cavalli A., Walsh P., Lundström P., Zarrine-Afsar A., Sharpe S., Vendruscolo M., Kay L. E. (2012). Structure of an intermediate state in protein folding and aggregation. Science.

[cit123] Feng H., Zhou Z., Bai Y. (2005). A protein folding pathway with multiple folding intermediates at atomic resolution. Proc. Natl. Acad. Sci. U. S. A..

[cit124] Tinzl M., Hilvert D. (2021). Trapping Transient Protein Species by Genetic Code Expansion. ChemBioChem.

[cit125] Zander T., Phadke N. D., Bardwell J. C. (1998). Disulfide bond catalysts in Escherichia coli. Methods Enzymol..

[cit126] Scheraga H. A., Wedemeyer W. J., Welker E. (2001). Bovine pancreatic ribonuclease A: oxidative and conformational folding studies. Methods Enzymol..

[cit127] Bucciantini M., Giannoni E., Chiti F., Baroni F., Formigli L., Zurdo J., Taddei N., Ramponi G., Dobson C. M., Stefani M. (2002). Inherent toxicity of aggregates implies a common mechanism for protein misfolding diseases. Nature.

[cit128] Haass C., Selkoe D. J. (2007). Soluble protein oligomers in neurodegeneration: lessons from the Alzheimer's amyloid beta-peptide. Nat. Rev. Mol. Cell Biol..

[cit129] Eisenberg D., Jucker M. (2012). The amyloid state of proteins in human diseases. Cell.

[cit130] Ninagawa S., Tada S., Okumura M., Inoguchi K., Kinoshita M., Kanemura S., Imami K., Umezawa H., Ishikawa T., Mackin R. B., Torii S., Ishihama Y., Inaba K., Anazawa T., Nagamine T., Mori K. (2020). Antipsychotic olanzapine-induced misfolding of proinsulin in the endoplasmic reticulum accounts for atypical development of diabetes. eLife.

[cit131] Creighton T. E., Darby N. J., Kemmink J. (1996). The roles of partly folded intermediates in protein folding. FASEB J..

[cit132] Nishino H., Kitamura M., Okada S., Miyake R., Okumura M., Muraoka T. (2022). Cysteine-based protein folding modulators for trapping intermediates and misfolded forms. RSC Adv..

